# High-Throughput Mass Spectrometry Analysis of *N*-Glycans and Protein Markers after *FUT8* Knockdown in the Syngeneic SW480/SW620 Colorectal Cancer Cell Model

**DOI:** 10.1021/acs.jproteome.3c00833

**Published:** 2024-03-20

**Authors:** Rubén López-Cortés, Laura Muinelo-Romay, Almudena Fernández-Briera, Emilio Gil Martín

**Affiliations:** † Doctoral Program in Methods and Applications in Life Sciences, Faculty of Biology, Universidade de Vigo, Campus Lagoas-Marcosende, 36310 Vigo, Pontevedra (Galicia), Spain; ‡ Liquid Biopsy Analysis Unit, Translational Medical Oncology (Oncomet), Health Research Institute of Santiago de Compostela (IDIS), CIBERONC, Travesía da Choupana, 15706 Santiago de Compostela, A Coruña (Galicia), Spain; § Molecular Biomarkers, Biomedical Research Centre (CINBIO), Universidade de Vigo, Campus Lagoas-Marcosende, 36310 Vigo, Pontevedra (Galicia), Spain; ∥ Nutrition and Food Science Group, Department of Biochemistry, Genetics and Immunology, Faculty of Biology, Universidade de Vigo, Campus Lagoas-Marcosende, 36310 Vigo, Pontevedra (Galicia), Spain

**Keywords:** *FUT8*, core fucosylation, colorectal cancer, glycomics, proteomics, mass spectrometry, profiling

## Abstract

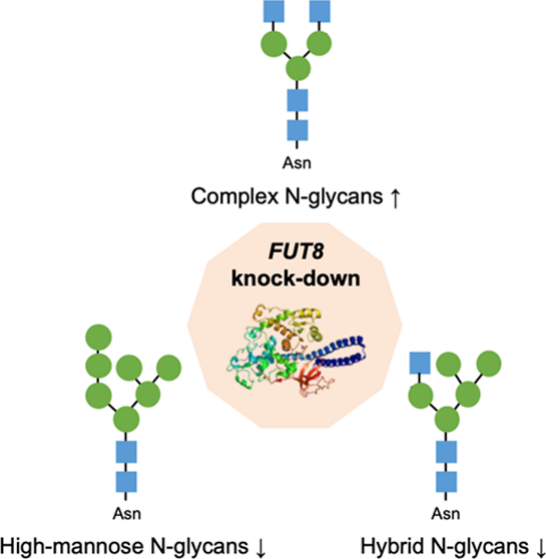

Disruption of the glycosylation machinery is a common feature in many types of cancer, and colorectal cancer (CRC) is no exception. Core fucosylation is mediated by the enzyme fucosyltransferase 8 (FucT-8), which catalyzes the addition of α1,6-l-fucose to the innermost GlcNAc residue of *N*-glycans. We and others have documented the involvement of FucT-8 and core-fucosylated proteins in CRC progression, in which we addressed core fucosylation in the syngeneic CRC model formed by SW480 and SW620 tumor cell lines from the perspective of alterations in their *N*-glycosylation profile and protein expression as an effect of the knockdown of the *FUT8* gene that encodes FucT-8. Using label-free, semiquantitative mass spectrometry (MS) analysis, we found noticeable differences in *N*-glycosylation patterns in *FUT8*-knockdown cells, affecting core fucosylation and sialylation, the Hex/HexNAc *ratio*, and antennarity. Furthermore, stable isotopic labeling of amino acids in cell culture (SILAC)-based proteomic screening detected the alteration of species involved in protein folding, endoplasmic reticulum (ER) and Golgi post-translational stabilization, epithelial polarity, and cellular response to damage and therapy. This data is available via ProteomeXchange with identifier PXD050012. Overall, the results obtained merit further investigation to validate their feasibility as biomarkers of progression and malignization in CRC, as well as their potential usefulness in clinical practice.

## Introduction

Colorectal cancer (CRC) is the third most common cancer in both sexes and also the third most prevalent cause of cancer death in the developed world.^1^ In 2020, more than 1.9 million diagnoses and more than 930,000 deaths were recorded. Three major, nonmutually exclusive regulatory pathways share the complex molecular etiology of CRC: chromosomal instability (CIN), which accounts for 85% of all cases and exhibits the hallmark of *APC* loss, along with microsatellite instability (MSI) and the CpG island methylator phenotype (CIMP) responsible for the remaining cases.^2^ Colorectal tumors are therefore heterogeneous,^3^ and it is common for many entities to share the features of several carcinogenic pathways.^2^

The currently available high-throughput methodology reveals the molecular complexity of colorectal tumors. Massive parallel sequencing methodologies can thus be used to detect myriad (epi)genetic and susceptibility *loci* alterations that, gradually accumulating over 10 to 15 years, give rise to colorectal mucosa cancerization and the subsequent invasiveness and spread of CRC.^4^ However, the mutational sequences of CRC-determining genes, such as *KRAS*, *APC*, or *TP53*, are still under discussion.^5^ For their part, high-throughput proteomic platforms hold the promise of revealing the proteins that drive the malignant evolution of colorectal cells. In this regard, mass spectrometry (MS)-based proteomics facilitates comprehensive and systematic protein signatures for the accelerated profiling of putative diagnostic and prognostic biomarkers. Furthermore, MS technology enables us to tackle glycoconjugate microheterogeneity, overcoming the limitations of alternatives such as lectin blots or lectin arrays.^6^ Thus, proteomic and glycomic facilities currently allow large-scale sample analysis, quickly and accurately determining several thousand protein or glycan species in a single run. It is even possible to quantify specimens of interest, either absolutely through isotopic labeling or relatively using label-free approaches.^7−10^ It is worth noting that several studies have addressed the viability of MS for profiling the CRC glycome, revealing specific patterns associated with clinicopathological stages, tumor progression, or response to treatment.^11−13^ The contributions of proteomic-derived signatures in this context are also substantial.^14−17^ MS performed on cell samples has the strength to assess the effects on protein expression and/or glycosylation after homeostasis is challenged by drugs, gene disruption, or any other perturbation. However, although in vitro cell culture is an excellent experimental approach, it lacks the cellular heterogeneity of tumors, which is why more realistic models of the tissue microenvironment, such as three-dimensional (3D) cell cultures of spheroids and organoids, are progressively being adopted.^18−20^

*FUT8* encodes the fucosyltransferase 8 (FucT-8) enzyme, responsible for core fucosylation, a key modification of the *N*-glycan core involving the transfer of GDP-l-Fucose to the innermost GlcNAc via α(1,6)linkage. In CRC, as well as in other cancer types, several proteins have been reported as aberrantly core fucosylated.^21,22^ Thus, the expression and activity of FucT-8 directly affect the pool of core-fucosylated proteins, although the *statu quo* of FucT-8 and the level of core fucosylation at different stages of the tumor cycle are hard to predict. For example, whereas in gastric cancer, decreased core fucosylation contributes to malignancy,^23^ in lung tumors, FucT-8 overexpression is associated with a worse outcome.^24^ Regarding CRC, our group first addressed the expression and activity of FucT-8 in CRC tissue and premalignant lesions.^25−27^ We found that the upregulation of FucT-8 was related to the degree of tumor infiltration, hypothesizing the involvement of the enzyme in carcinoma progression.^27^ We then developed a core-fucosylation-deficient cellular system in the syngeneic CRC lines SW480 and SW620 by shRNAi-knockdown of the *FUT8* gene.^28,29^ Using this model, we found that FUT8 knockdown in the primary tumor cell line SW480 led to a more mesenchymal phenotype, with increased proliferation and reduced migration and adhesion,^28^ as well as enhanced sensitivity to TRAIL-induced apoptosis.^29^ Therefore, the role of FucT-8 in CRC remains somewhat uncertain, as a combination of pro-tumor and tumor-suppressive effects have been observed. Nevertheless, FucT-8 seems to be dynamically modulated depending on the tumor stage since FUT8 knockdown in metastatic SW620 cells generally caused no effects or minor phenotypic repercussions compared to the nonmetastatic syngeneic SW480 line.

To contribute to the understanding of how FucT-8 and core fucosylation mediate CRC progression, our aim in the present study was to scrutinize the *N*-glycome and proteome of the SW480/SW620 shFUT8 CRC model. By comparing *N*-glycans in SW480 and SW620 CRC cells, as well as in their respective *FUT8*-knockdown clones, we found that FucT-8 depletion affected the microheterogeneity of *N*-glycans, leading to greater complexity in the equilibrium of *N*-glycoproteins, and potential differences in their behavior. Interestingly, FucT-8 activity also affected the expression of nonglycosylated proteins, some of them related to endoplasmic reticulum (ER) stress or cell polarity, which may be relevant in terms of response to therapy and cell phenotype. In summary, the results contribute to an improved molecular description of CRC, are in line with previous findings by ours and other groups, and together point to FucT-8 inhibition as a potential target for CRC therapy.

## Materials and Methods

### Cell Culture

Caco2, HCT116, and the syngeneic CRC lines SW480 and SW620 were obtained from the ATCC (American Type Culture Collection) and were kindly donated by the Health Research Institute of Santiago de Compostela (IDIS, Spain). Wild-type cells were maintained in DMEM (Sigma-Aldrich) supplemented with 10% FBS (Life Technologies) and 10,000 U/mL penicillin–streptomycin (Life Technologies) and maintained at 37 °C in a humidified incubator supplied with 5% CO_2_. The medium for *FUT8* knockdown and nontransfected control (NTC) cells was supplemented with 5 μg/mL puromycin (Sigma-Aldrich). *FUT8* knockdown and NTC clones were obtained by lentiviral transfection and ulterior lectin selection, as previously published by our research team.^28^ Briefly, *FUT8*-knockdown clones (F52L and F59L) were isolated by supplementing the growth medium with 500 μg/mL of *Lens culinaris* agglutinin (LCA) for 7 days; the cells were then seeded in the medium without LCA. Consequently, the biological material for the experimental work was made up of eight cell lines: SW480 F52L, SW480 F59L, SW620 F52L, SW620 F59L, and their corresponding controls (SW480 NTC and SW620 NTC).

### Enzymatic Hydrolysis of the *N*-Glycosidic Bond with PNGase F

Cell extracts (∼2 × 10^6^ cells) from three biological replicates of the HCT116 line and SW480/SW620 cells were resuspended in 100 μL of mQ water and sonicated in a water bath for 30 min to promote complete cell lysis. The glycans were subsequently released using an adapted working protocol.^30^ In detail, aliquots of the sonicated pellets were diluted with denaturing buffer (5.8 M GuHCl, 5 mM DTT) such that 2.5 × 10^5^ cells were loaded into each well of a HTS 96-well plate preconditioned with a hydrophobic Immobilon-P PVDF membrane on the bottom. All of the cell samples were loaded in duplicate before incubating the plates in a humidified oven at 60 °C. After incubation, the plates were shaken horizontally for 5 min before being centrifuged at 500*g* for 1 min. Each well was then washed twice with 200 μL of mQ water and once with 200 μL of 100 mM NaHCO_3_, with ∼2 min incubations on a horizontal shaker between washes.

In-well enzymatic hydrolysis of glycans was performed in a 50-μL solution of 100 mM NaHCO_3_ and 1 mU of PNGase F (Roche Life Sciences). Finally, the plates were incubated overnight at 37 °C and then collected by centrifugation (500*g*, 2 min).

### Derivatization of *N*-Glycans and HILIC-SPE

The released *N*-glycans were derivatized by an adaptation of the ethyl esterification protocol^31^ that stabilizes sialic acids and allows differentiating α(2,3)- from α(2,6)-sialylation due to the different molecular mass of the fragments to be obtained by mass spectrometry. For this purpose, 20 μL of the *N*-glycan solution plus 100 μL of ethyl esterification reagent (0.25 M EDC and 0.25 M HOBt, 1:1 v/v) were incubated for 1 h at 37 °C. Then, 100 μL of ACN was added and the solution was incubated at −20 °C for 15 min. At this point, the sialic acids of the *N*-glycans were derivatized and could be extracted from the reaction mixture.

The purification was performed using cotton HILIC-SPE microtips^32^ using 20-μL pipet microtips hand-packed with thin cotton strands. The columns were equilibrated with 3 washes of 20 μL of mQ water and conditioned with 3 washes of 20 μL of an 85% ACN solution. Subsequently, the *N*-glycans were extracted from the solution by continuous pipetting of the derivatization mixture. The pipet microtips were then washed by 3 cleaning cycles with 20 μL of 85% ACN/1% TFA and another 3 cycles with 20 μL of 85% CAN. As a final step, *N*-glycans were eluted by repeatedly up-and-down pipetting with 10 μL of mQ water.

### Analyzing Derivatized *N*-Glycans Using MALDI-TOF MS

For matrix-assisted laser desorption/ionization-time of flight mass spectrometry (MALDI-TOF) analysis, 5 μL of ethyl-esterified derivatized *N*-glycans was deposited on a MALDI plate (Bruker Daltonics), followed by the addition of 1 μL of a 1-mg/mL super-DHB solution in a mixture of ACN/mQ (1:1, v/v, Sigma-Aldrich) and 1 mM NaOH on this drop. The samples were allowed to dry completely until crystallization before the plate was inserted into the mass spectrometer. MALDI-TOF spectra were acquired on an UltrafleXtremeTM mass spectrometer in positive reflectron mode activated and controlled by FlexControl 3.4 Build 119 software (Bruker Daltonics). The apparatus was calibrated using the Bruker peptide kit in a working window of 1000 to 5000 *m*/*z* and ion suppression set at 900 *m*/*z*. A total of 10,000 shots were fired at a frequency of 1000 Hz, and they were grouped into batches of 200 shots fired randomly over the spot region. Where necessary, MALDI-TOF/TOF MS fragmentation was performed to obtain complementary information for the structural elucidation of the signals of interest.

### Processing *N*-Glycan Spectra Data Using MALDI-TOF MS

For each cell line, 3 protein extracts were analyzed, each of them replicated 4 times. The MS spectra were analyzed in the proprietary computer script known as MassyTools v0.1.5.1, whose code was developed in Python 2.7.3 language (Python Software Foundation; http://docs.python.org/py3k/reference/index.html). This set of spectra was recalibrated internally using glycans of known unique composition as standards. Those signals with a correct isotopic distribution (0.95), an S/N ratio greater than 2, and a shift window of ±20 ppm were selected for structural analysis using the Glyco-Peakfinder tool (GlycoWorkbench 2.1; http://www.eurocarbdb.org/), which generated their glycosidic structures, unique or all possible considering the species being worked (*Homo sapiens*). For the selected *N*-glycan species, we then further confirmed whether their proposed structures matched the MS/MS signals. The intensity of the signals that passed the filters described above was normalized by relative quantification as (signal intensity/sum of signals in the spectrum) × 100.

To observe changes in the *N*-glycosylation pattern of the cell lines under study, the normalized signals were grouped according to the types of *N*-glycans commonly named in the literature, as well as by traits of interest. These groupings were defined by a number whose biological meaning is a percentage average, i.e., it indicates the percentage of *N*-glycosidic structures that meet the grouping criteria.

Table 1 lists these classificatory groupings, as well as the mathematical formula used in each case to make the selection and calculate the sum of standardized intensities that meet the requirements. The signals were first classified as mannose-rich *N*-glycan, paucimannosidic, complex or hybrid structures; second, we evaluated the presence of monofucosylation, multifucosylation, α(2,3)- and α(2,6)-sialylation, and fucosylation with concomitant α(2,3)-sialylation; third, we assessed the Hex/HexNAc ratio; finally, the number of antennas was also evaluated.

**Table 1:** *N*-Glycan Structure Classification and Formulas for Derived Trait Calculations for MS Signals^a^

*N*-glycan classification	calculation (in relative intensity total 100%)
by type:	
paucimannosidic	
rich in mannose	
complex	
hybrid	
by glycidic motif:	
monofucosylation	
multifucosylation	
α(2–3)-sialylation	
α(2–6)-sialylation	
mixed sialylation	
fucosylation and α(2–3)-sialylation [sLe^x^]	
by H/N ratio:	
Hex > HexNac	
Hex = HexNac	
Hex < HexNac	
by antennarity:	
mono-	
di-	
di-/tri-	
tri-	
tri-/tetra-	
tetra-	
tetra-/poly-	
poly-	

a Hex = Hexose; HexNac = *N*-acetylhexosamine; ENeuAc = α(2,6)-*N*-acetylneuraminic acid; LNeuAc = α(2,3)-*N*-acetylneuraminic acid; Fuc = fucose. Note: The presence of poly-LacNAc (*N*-acetyl-lactosamine) structures is exclusively considered in polyantennarity.

### Stable Isotopic Labeling of Amino Acids in Cell Culture (SILAC)

DMEM high glucose provided by Dundee Cells was chosen as the culture medium to keep cell growth conditions as unchanged as possible in comparison to other assays. R6K6 (light, L) and R10K8 (heavy, H) alternatives were used. Wild-type (wt) SW480, *FUT8*-knockdown clones SW480 F52L and SW480 F59L, and SW620 NTC cells were tagged using the L medium. Likewise, SW620 wt, *FUT8*-knockdown clones SW620 F52L and SW620 F59L, and SW480 NTC cells were tagged using the H medium. The labeled lysines and arginines were incorporated by maintaining the cell culture in Petri dishes for a minimum of 1 month (8 passages in total). At the end of the adaptation phase, cell numbers were increased by transferring the culture to BD Falcon Multi-Flask 5-deck plates (875 cm^2^) until ≈10^7^ cells were reached.

### Protein Prefractionation and Enrichment

**Figure 1:**
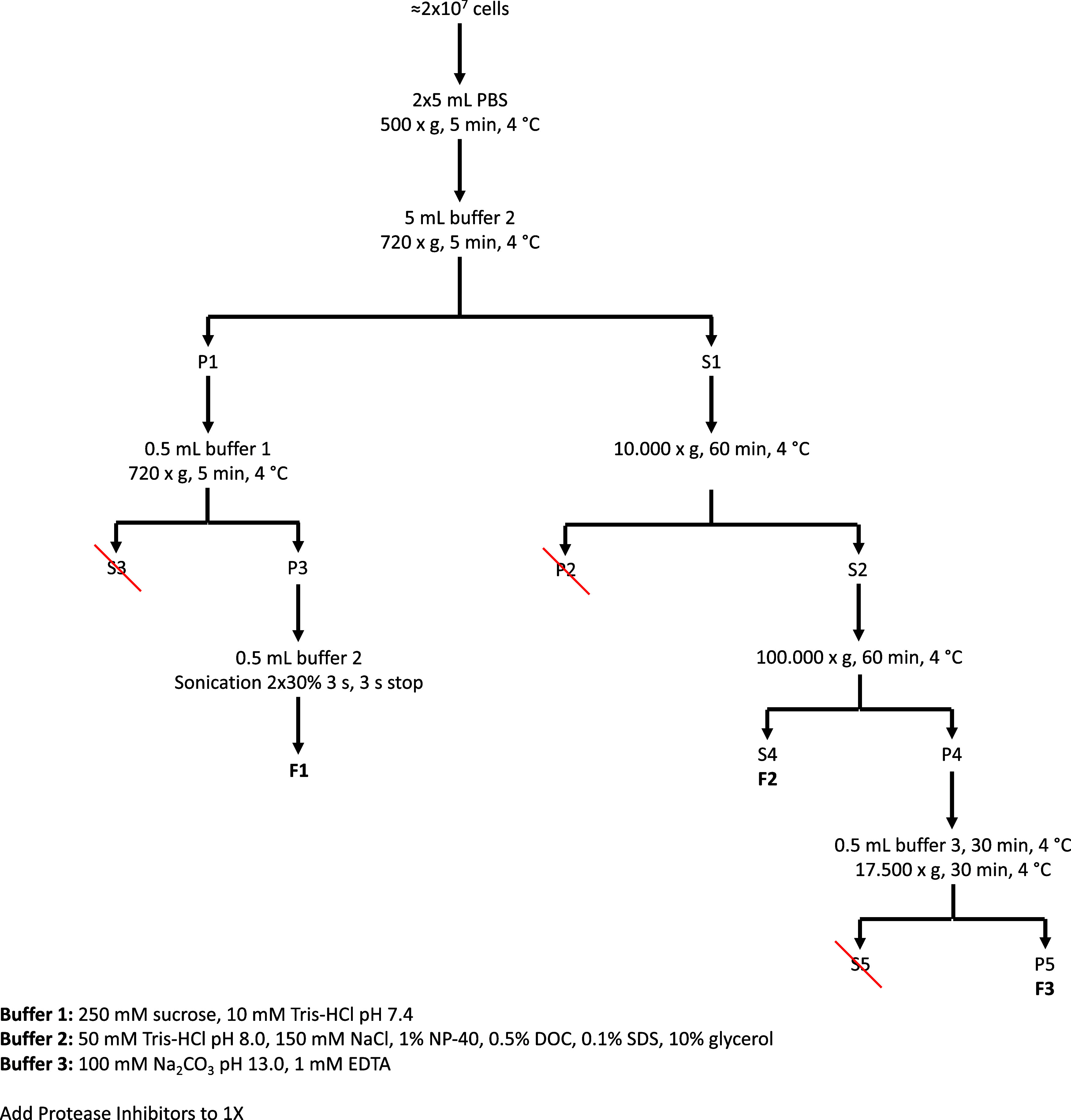
Flowchart depicting the ultracentrifugation protocol to obtain the enriched protein fractions used for MS analysis. F1: proteome fraction 1, F2: proteome fraction 2, and F3: proteome fraction 3.

High centrifugal forces over long periods allow the organelles and macromolecules in the cells to sediment. In this regard, differential centrifugation accompanied by buffers of different compositions can disaggregate the organelles sequentially so that it is possible to separate their protein content. In our case, the fractionation protocol included detergents, such as NP-40, deoxycholic acid (DOC), or sodium dodecyl sulfate (SDS), capable of disrupting the cell membrane without substantially affecting the rest of the organelles, as well as solubilizing its proteins due to their higher hydrophobicity compared to the surface agents of the cytosol.^33^ The workflow (Figure 1) started with cell suspensions (≈2 × 10^7^ intact cells), which were washed with PBS and resuspended in 5 mL of buffer 2 (50 mM Tris–HCl (pH 8.0), 150 mM NaCl, 1% NP-40, 0.5% DOC, 0.1% SDS, 10% glycerol). Subsequently, the cells were gently centrifuged at 720*g* for 5 min at 4 °C. The first pellet (P1) was resuspended in 500 μL of buffer 1 (250 mM sucrose, 10 mM Tris–HCl (pH 7.4)) and centrifuged for another 5 min at 720*g*. The new pellet (P3) was dissolved in 500 μL of buffer 2 and sonicated twice at 30% for 3 s, with a 3 s interval between cycles. On the other side, the first supernatant (S1) was centrifuged at 10,000*g* for 1 h at 4 °C to obtain a clean supernatant (S2), which was then ultracentrifuged at 100,000*g* for 1 h at 4 °C. The final supernatant (S4) was labeled as fraction 2 (F2), while the pellet (P4) was resuspended in 500 μL of buffer 3 (100 mM Na_2_CO_3_ (pH 11. 3), 1 mM EDTA) and centrifuged at 17,500*g* for 30 min at 4 °C. The final pellet (P5) corresponded to the protein fraction 3 (F3) used for proteomic analysis by mass spectrometry. All buffers were supplemented with a protease inhibitor tablet (Roche Life Sciences) to prevent protein degradation.

### Gel Electrophoresis

One hundred micrograms of protein from cell lysates from the protein fraction F3 was reconstituted in 20 μL of pH 7.4 buffer (0.2 M Tris–HCl, 2% w/v SDS, and 20% v/v glycerol) and mixed with 4 μL of SDS-PAGE loading buffer (10% w/v SDS, Tris-Base 40 mM (pH 6.8), 50% v/v glycerol, 0.1% v/v bromophenol blue, 10% v/v β-mercaptoethanol). The samples were then denatured by heating at 100 °C for 5 min and loaded onto a 0.75 mm thick discontinuous gel composed of a 10% acrylamide/bis-acrylamide stacking gel and a 12.5% acrylamide/bis-acrylamide running gel. Separation was carried out at 180 V (constant voltage) for 10 min or until the electrophoretic front completely penetrated the running gel. Subsequently, the gel was fixed for 30 min in 40% (v/v) ethanol and 10% (v/v) acetic acid and then stained overnight with Coomassie Blue R-250 (Bio-Rad). Finally, the gels were rinsed with distilled water until a clear background was observed.

### In-Gel Trypsin Protein Digestion

Electrophoresis bands were manually excised and transferred to 2.5 mL protein LoBind tubes (Eppendorf) and then washed twice with distilled water and 50% (v/v) ACN/25 mM AmBic until no trace of blue color was observed. Afterward, the gel spots were washed with 25 mM AmBic and dehydrated with pure ACN. Fifty microliters of 20 mM DTT (Bio-Rad) in 25 mM AmBic was added for 1 h at 37 °C. After this time, the solution was removed and washed with 100 μL of 25 mM AmBic. Again, 100 μL of ACN was added, and once the gel turned white, it was removed. Next, 50 μL of 100 mM iodoacetamide (IAA, Bio-Rad) in 25 mM AmBic was added and allowed to react in the dark at room temperature for 45 min. Finally, the pellets were washed repeatedly with 25 mM AmBic/50% ACN solution and dried with 100% ACN.

For protein digestion, 30 μL of trypsin (20 ng/μL in 12.5 mM AmBic/2% (v/v) ACN) was added to the gel spots and incubated for 60 min at 0 °C. The nonabsorbed trypsin solution was subsequently removed, and the gels were covered with 100 μL of 12.5 mM AmBic. The samples were incubated for 12 h at 37 °C, and then 50 μL of 5% (v/v) formic acid (FA) was added. The supernatant was transferred to a fresh LoBind tube, and the peptides were isolated using 3 consecutive 50% (v/v) ACN/0.1% (v/v) trifluoroacetic acid (TFA) extractions and a final wash with ACN. The samples were dried and stored at −20 °C until use.

### Protein Identification Using LC-ESI-LTQ Orbitrap MS and Data Analysis

The protein extracts corresponding to the protein fraction 3 (Figure 1) were analyzed by MS at the Proteomics Unit of the Cancer Research Center of the University of Salamanca (CiC-USAL, Spain; center associated with the ProteoRed network, PRB2-ISCIII). MS spectra were obtained using an ESI-LTQ Orbitrap Velos ETD mass spectrometer (Thermo Fisher) coupled to an Acquity nano-HPLC (Waters) equipped with a Symmetry C18 precolumn (5 μm, 20 mm × 180 μm) and a BEH C18 column (1.7 μm, 75ID 25 cm), which was eluted with a 1–40% ACN/0.1 FA gradient (120 min). Sample aliquots of 5 μL (0.15 μg/μL) were injected, and higher-energy collision dissociation (HCD) and electron transfer dissociation (ETD) fragmentation patterns were obtained to ensure the highest quality results. The spectra data were processed using MaxQuant v1.5.6.5, Perseus v1.5.6.0, and the UniProt database. The isotopic incorporation rate was calculated using the formula of mean incorporation (%) = 1 – (1/(mean + 1)) × 100.^34^ Furthermore, as the medium was not supplemented with proline, the data is expected to show an interconversion of arginine to proline. The interconversion was determined using MaxQuant to be 7.7%, and this value was used to correct the proteome quantification data. Analyses of the interactional networks of functional proteins were performed using STRING (protein–protein interaction networks functional enrichment analysis) v.10.0 (http://string-db.org) and protein analysis through evolutionary relationships (PANTHER) v.17.0 (http://www.pantherdb.org) databases.^35,36^ The mass spectrometry proteomics data have been deposited to the ProteomeXchange Consortium via the PRIDE partner repository with the data set identifier PXD050012.

### Statistical Analysis

Preprocessed cell *N*-glycome MS data was imported into SIMCA software Version 13.0 (Umetrics AB) in order to perform a principal component analysis (PCA) to reveal outliers and batch effects. The cell line samples were displayed in the score plots, while the relative intensity values for each glycan were displayed in the loading plots. The samples located on the peripheries of the score plots showed a large deviation from the other samples, and they were removed if they fell outside the borders. The relative intensities of glycan-derived traits were calculated (paucimannose, high-mannose, hybrid, and complex type). We then calculated the additional glycan-derived traits (fucosylation, sialylation, Hex/HexNAc ratio, and potential number of antennae). When the total sum was greater than 100%, it indicated nonunivocal structures whose signal can be assigned to at least two groups of *N*-glycans. To explore the differences in glycan traits, the Student’s *t*-test was performed using IBM SPSS Statistics v26 software. Statistical significance was set at (*) *p* ≤ 0.05 and (**) *p* ≤ 0.01. Microsoft Excel 2013 was used to represent the data graphically.

The cell proteome data filter at the 1% FDR was used to calculate the SILAC heavy:light ratios in MaxQuant v1.5.6.5, correcting the peptide SILAC heavy:light ratios for arginine–proline interconversion by the formula *r*[c] = *r*[o]/((1 – *p*)*^n^*), where *r*[c] is the corrected ratio, *r*[o] is the observed ratio, *p* is the conversion rate, and *n* is the number of proline residues per peptide. The protein ratios were then calculated using the median of the peptide ratios. With Perseus, statistical tests could not be performed, as there was a lack of replicates. However, an exploratory analysis was performed using numeric Venn diagrams. There were a total of 963 proteins identified with two or more unique peptides, and a total of 190 proteins quantitated.

## Results

### Rationale of MS-Based Characterization and Semiquantificative Separation of *N*-Glycome from the SW480/SW620 shFUT8-Knockdown CRC Model

The method to screen the *N*-glycome of the SW480/SW620 shFUT8-knockdown CRC model consisted of three steps. First, the cell extracts were solubilized with chaotropic agents and adsorbed onto PVDF membranes.^30^ Second, the oligosaccharide chains were digested with PNGase F and subsequently derivatized by ethyl esterification in order to stabilize sialylation and to differentiate α(2,3)- from α(2,6)-sialylation.^31^ Third, mixtures of derivatized *N*-glycan were purified in cotton HILIC-SPE for convenient analysis by MALDI-TOF MS.^32^ A total of 8 CRC lines belonging to our syngeneic cellular model SW480/SW620 shFUT8^28^ were analyzed: SW480/SW620 wt, SW480/SW620 NTC, SW480/SW620 F52L, and SW480/SW620 F59L clones. In addition, the CRC HCT116 cell line, which lacked the GDP-mannose-4,6-dehydratase enzyme,^37^ was used as a negative control for fucosylation.

A PCA model based on technical replicates was generated using SIMCA software. This resulted in a model explaining 72.4 and 77% (R2Xcum) of the data from SW480 and SW620 cells, respectively, as well as a good prediction power of 60 and 65.7% (Q2cum). By coloring the scores according to replicates, a clear overlap was found (Figure S1A), indicating the robustness of the model for assessing the glycosylation characteristics of the SW480/SW620 shFUT8 CRC cell model. Spots that were exceptionally clustered outside the Hotelling’s T 95% ellipse were discarded in subsequent MALDI-TOF MS analyses. Taking advantage of clonal selection with LCA, we compared the glycomic composition of silenced SW480 and SW620 cells (their respective clones F52 and F59) with that of those silenced and treated with LCA (clones F52L and F59L). Figure S1B,C shows the high degree of overlap between the wt and NTC cells of both lines, while the replicates of the *FUT8*-knockdown clones, with and without LCA selection, clustered together, except for the clone SW620 F59, which showed a high degree of dispersion (Figure S1C). These findings reinforced the benefits of including LCA selection in the generation of *FUT8*-knockdown clones.^28^ Correspondingly, the following MALDI-TOF MS glycome analyses were carried out using the *FUT8*-silenced LCA-treated cells.

**Figure 2:**
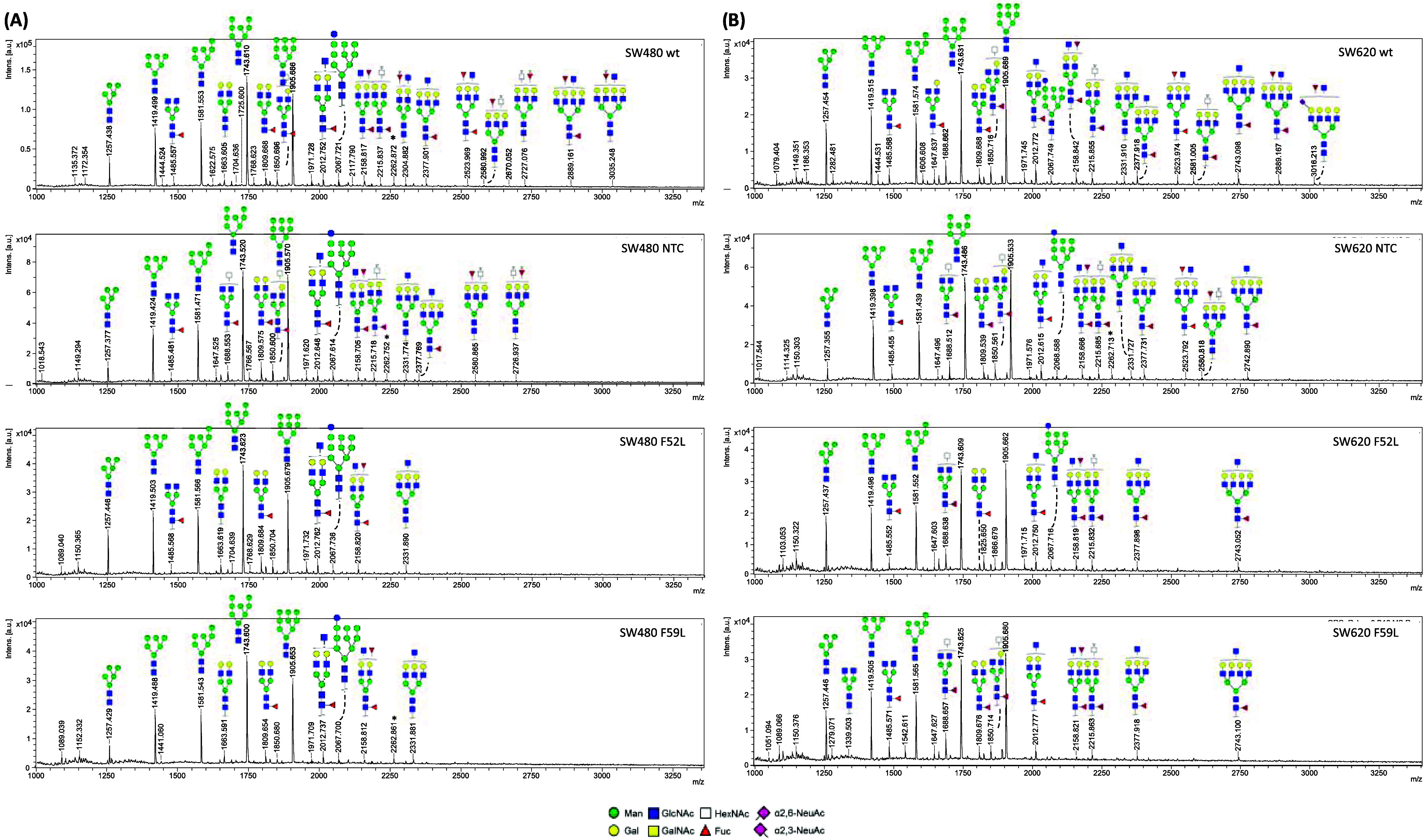
Representation of typical MS spectra from (A) SW480 *N*-glycans and (B) SW620 *N*-glycans. The most probable oligosaccharide structure that can be assigned to each MS signal is plotted on top of the corresponding MS peak signal. Annotation was performed using GlycoWorkbench 2.1 and the SNFG notation: Symbol Nomenclature For Glycans (SNFG)—NCBI. Available at: https://www.ncbi.nlm.nih.gov/glycans/snfg.html.

Each spot deposited on the MALDI plate provided an MS spectrum with a set of *m*/*z* signals that can be related to specific oligosaccharide structures or, if no tandem MS/MS (MS2) fragmentation is performed to elucidate the exact structure, with two or three indistinguishable possibilities. In this sense, Figure 2A shows representative MS spectra for SW480 cells, and Figure 2B shows SW620 cells, indicating the most likely oligosaccharide structures that could correspond to each MS signal. Additionally, Figure S2A,B shows the MS2 spectra obtained from the fragmentation of the 1809.504 and 1982.482 *m*/*z* signals, respectively, which are two representative examples of how to study the MS fragmentation pattern to infer the presence or absence of certain saccharide residues by analyzing the loss of specific *m*/*z* values. Thus, a loss of 146.06 Da produced a peak at 1663.420 Da, compatible with the presence of fucose. Similarly, the loss of 319.13 Da produced an intense signal at 1,663,142 Da that is consistent with the presence of a derivatized α(2,6)-sialic acid attached to a terminal galactose. However, instead of manually fragmenting each MS signal, we conducted an internal calibration control with the peaks of univocal assignment and intense signal in order to use the automatic structure assignments provided by Glyco-Peakfinder software when entering the internal calibration signals.

This internal calibration allowed us to adjust the rest of the signals with MassyTools v0.1.5.1 software and subsequently perform a screening to keep only those signals with a correct isotopic distribution (0.95), an S/N ratio higher than 2 and a shift window of ±20 ppm with which to study the *N*-glycome profile listed in Figure S3. This semiquantitative, label-free approach assumed that the value of the area under the curve (AUC, the result of subtracting the background of each signal, normalizing its value with respect to the total sum of all of the AUC of each spectrum, and expressing the result as a percentage) can be valid for comparing the expression of *N*-glycosidic structures corresponding to *m*/*z* signals in the different lines of our SW480/SW620 sh*FUT8* CRC cell model. In summary, we have identified a total of 299 *m*/*z* peaks in the whole set of MS spectra from SW480 and SW620 cells, of which 206 have exceeded the signal-to-noise >2 thresholds in at least one of the replicates for the SW480 set and 193 for SW620. From this selection, 236 and 226 possible *N*-glycome structures could be proposed for the SW480 and SW620 line sets, respectively (Supporting Information 1). Then, we proceeded to evaluate the relative abundances of each *N*-glycan type according to the derived trait calculations depicted in Table 1, which reinforced the robustness and quality of our assignments.

### MALDI-TOF MS Profiling of the *N*-Glycome from the SW480/SW620 shFUT8-Knockdown CRC Model

**Figure 3:**
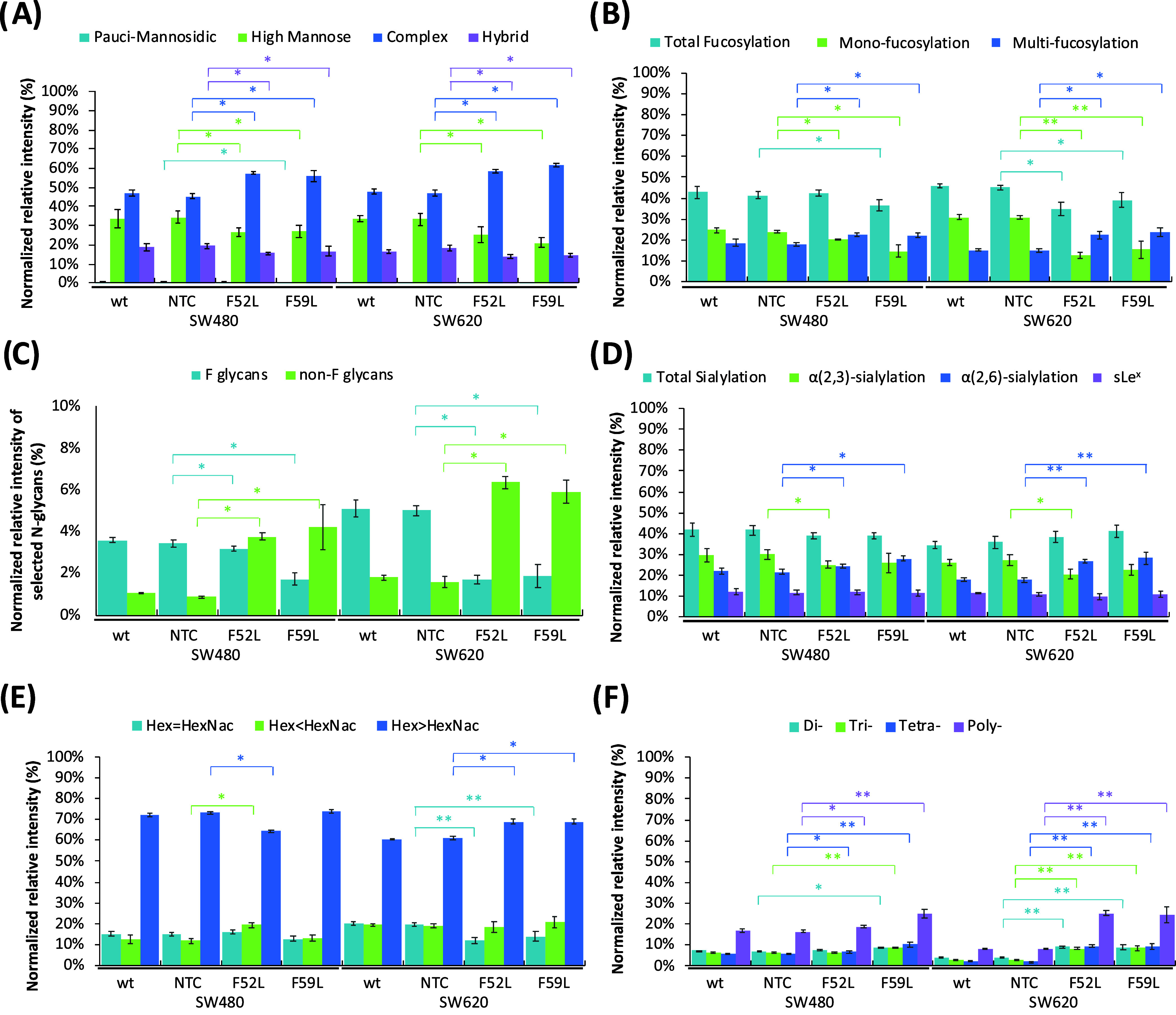
Relative quantification of derived glycan traits according to glycan classes: (A) *N*-glycan types; (B) fucosylation distinguishing monofucosylated (1 fucose) and multifucosylated (>2 fucoses) structures; (C) details of selected *N*-glycans, with group F(ucosylated) consisting of the signals at *m*/*z* 1485.533 (H3N4F1), 1647.586 (H4N4F1), 1809.639 (H5N4F1), 1850.666 (H4N5F1), 2012.719 (H5N5F1), 2174.771 (H6N5F1), and the corresponding non-F(ucosylated) versions at *m*/*z* 1339.476 (H3N4), 1501.529 (H4N4), 1663.581 (H5N4), 1704.608 (H4N5), 1866.661 (H5N5), and 2,028.714 (H6N5); (D) *N*- glycans with mixed sialylation, as well as α2,3-, α2,6-sialylated *N*-glycans; (E) hexose (Hex)/*N*-acetylhexosamines (HexNAc) ratios; (F) antennarity of *N*-glycans. Error bars show the standard deviation between 3 replicates. When the sum is greater than 100%, it means that there are nonunivocal structures whose signal can be assigned to at least two groups of *N*-glycans. *p < 0.05 and **p < 0.01 according to the Student’s *t*-test.

As can be seen in Figure 3A, the glycomic profile of the SW480/SW620 sh*FUT8* cell model was mostly dominated by complex *N*-glycans (normalized relative intensity: from 45.2 to 62.5%), followed by high-mannose *N*-glycans (18.1–34.5%) and hybrid *N*-glycans (14.3–19.4%). Paucimannosidic structures were in the minority (0.24–1.1%). In addition to being the most abundantly expressed type of *N*-glycan in the SW480/SW620 sh*FUT8* CRC cell model, the highest expression changes were also observed among the complex *N*-glycans from lines SW480 and SW620. Specifically, a significant increase in the expression of complex *N*-glycans was recorded in the *FUT8*-attenuated clones (Figure 3A). In the case of the SW480 group, expression ranged from 45.3 ± 2.0% in SW480 NTC to 57.4 ± 1.8% in SW480 F52L and 56.0 ± 2.5% in SW480 F59L (*p* < 0.05 for both comparisons according to the Student’s *t*-test), similar to that observed in the SW620 group, in which the expression ranged from 46.7 ± 1.5% in SW620 NTC to 58.3 ± 2.8% and 61.8 ± 2.1%, respectively (*p* < 0.05 from the Student’s *t*-test). In the case of hybrid *N*-glycans, expression was significantly reduced in the set of *FUT8*-attenuated clones (SW480/SW620 F52L and SW480/SW620 F59L) with respect to their NTC counterparts (Figure 3A). Indeed, the expression levels ranged from 19.5 ± 1.3% in SW480 NTC to 15.5 ± 0.6 and 16.7 ± 2.7% in F52L and F59L, respectively (*p* < 0.05 for both comparisons according to the Student’s *t*-test). Similarly, expression in the SW620 line varied from 18.1 ± 1.6% in SW620 NTC to 13.8 ± 1.1 and 14.3 ± 0.9% in F52L and F59L clones, respectively (*p* < 0.05 in both cases according to the Student’s *t-*test). This decreasing trend was also observed for high-mannose *N*-glycans (Figure 3A). Thus, 34.5 ± 3.0 and 33.3 ± 3.2% of high-mannose were detected in SW480 NTC and SW620 NTC, respectively, while their attenuated clones showed levels of 26.6 ± 2.4% (SW480 F52L) and 26.9 ± 3.0% (SW480 F59L), and 25.3 ± 3.9% (SW620 F52L) and 21.1 ± 2.7% (SW620 F59L), with all comparisons being statistically significant according to the Student’s *t*-test (*p* < 0.05, Figure 3A). Finally, a slight and statistically insignificant decrease was found in the paucimannosidic structures (Figure 3A) with the sole exception of the SW480 F59L clone (*p* < 0.05 from the Student’s *t*-test). Their respective expression levels were 0.6 ± 0.1% (SW480 NTC), 0.5 ± 0.1% (SW480 F52L), and 0.3 ± 0.1% (SW480 F59L). On the other hand, in the clones derived from the SW620 line, the expression remained constant (∼0.3 ± 0.1%). In summary, *FUT8* attenuation in the CRC cell model formed by the SW480 and SW620 lines led to an increase in the expression of the complex *N*-glycans to the detriment of hybrid and high-mannose *N*-glycans, while the expression of paucimannosidic structures remained essentially unchanged.

For the semiquantitative label-free analysis of fucosylated *N*-glycans, we selected MS signals with *m*/*z* compatible with the presence of the fucose residue, which introduces an increase of 146.06 Da that differs from that provided by other hexoses (+162.06 Da) or *N*-acetylhexosamines (+203.08 Da). With this aim, the MS signals were subdivided into two groups according to the existence of a single fucose residue in the structure (F1, monofucosylated *N*-glycans) or two or more fucoses (F, multifucosylated *N*-glycans) (Figure 3B). From a biochemical point of view, it can be assumed that the F1 group corresponded to the potential presence of a core fucose, especially if we rely on the Glyco-Peakfinder proposals, although this is not the only possible assignment and, therefore, it cannot be confirmed without corroboration via signal fragmentation using MS2. Conversely, signals within the multifucosylation F group would be indicative of the presence of terminal Lewis-type antigens. Taking this approach into account, we first observed that total fucosylation (i.e., both mono- and multifucosylated *N*-glycans) was statistically lower (*p* < 0.05 according to the Student’s *t*-test) in the silenced clones of the SW480 and SW620 lines than in their corresponding NTC controls, except for SW480 F52L. The convenience of disaggregating the fucosylated structures into F1 and F species was proven in that the suppression of *FUT8* led to opposite trends in the SW480 and SW620 lines. Thus, while monofucosylation was reduced in all silenced clones [24.0 ± 0.8% (SW480 NTC) vs 20.2 ± 0.5% (SW480 F52L) and 14.6 ± 2.8% (SW480 F59L); *p* < 0.05 according to the Student’s *t*-test, Figure 3B], multifucosylation was significantly increased [17.9 ± 1.0% (SW480 NTC) vs 22.5 ± 1.1% (SW480 F52L) and 22.2 ± 1.3% (SW480 F59L); *p* < 0.05, according to the Student’s *t*-test, Figure 3B]. A similar trend was observed in the SW620 clones, in which monofucosylation showed a statistically significant decrease [30.4 ± 0.9% (SW620 NTC) vs 12.4 ± 1.4% (SW620 F52L) and 15.3 ± 4.0% (SW620 F59L); *p* < 0.01, Figure 3B], while multifucosylation was statistically enhanced [14.8 ± 1.1% (SW620 NTC) vs 22.4 ± 1.9% (SW620 F52L) and 23.9 ± 2.1% (SW620 F59L); *p* < 0.05 according to the Student’s *t*-test, Figure 3B]. In addition, we selected 12 structures composed of the signals at *m*/*z* 1,485.533 (H3N4F1), 1647.586 (H4N4F1), 1809.639 (H5N4F1), 1850.666 (H4N5F1), 2012.719 (H5N5F1), and 2174.771 (H6N5F1), all of them carrying a core fucose, as well as their corresponding noncore-fucosylated versions at *m*/*z* 1339.476 (H3N4), 1501.529 (H4N4), 1663.581 (H5N4), 1704.608 (H4N5), 1866.661 (H5N5), and 2028.714 (H6N5) (Figure 3C). This subset of *N*-glycans allowed us to compare the simplest structures of diantennary [H3N4(F1), H4N4(F1), H5N4(F1)] and triantennary [H4N5(F1), H5N5(F1) and H6N5(F1)] *N*-glycans. All attenuated F52L and F59L clones, both SW480 and SW620, showed a decrease in species carrying the fucose residue along with a substantial increase in their equivalent afucosylated forms (Figure 3C).

As far as the presence of sialylation is concerned, we decided to start considering the label-free semiquantification of the entire set of sialylated structures, regardless of the bond type. In doing so, we found no apparent change in total sialylation levels in the SW480 and SW620 lines, with an overall average across the 8 lines studied of 39.0 ± 2.5% sialylated *N*-glycans (Figure 3D). However, the enzymatic release and the subsequent chemical derivatization led us to discriminate between α(2,3)- and α(2,6)-sialylation: α(2,3)Neu5Ac was detected by the presence of the signal of +273.08 Da due to lactonization, while α(2,6)Neu5Ac was characterized by an *m*/*z* signal of +319.13 Da. With respect to α(2,3)-sialylation, *FUT8*-attenuated F52L clones showed a statistically significant decrease [SW480 line: 30.0 ± 2.1% in NTC vs 25.1 ± 1.6%, *p* < 0.05, according to the Student’s *t*-test; SW620 line: 27.4 ± 2.7% in NTC vs 20.5 ± 2.4%, *p* < 0.05, according to the Student’s *t*-test; Figure 3D]. In contrast, α(2,6)-sialylation was statistically increased in both F52L and F59L clones of the SW40 line: 21.5 ± 1.2% (NTC) vs 24.6 ± 0.9% (SW480 F52L) and 27.9 ± 1.2% (SW480 F59L); *p* < 0.05, according to the Student’s *t*-test, Figure 3D. A similar change profile was observed for the SW620 line: 17.8 ± 1.1% (SW620 NTC) vs 26.6 ± 1.0% (SW620 F52L) and 28.3 ± 2.8% (SW620 F59L); *p* < 0.01, according to the Student’s *t*-test, Figure 3D. In conclusion, the attenuation of *FUT8* expression coincided with a decrease in the proportions of α(2,3)-sialylation in F52L clones and an increase in those of α(2,6)-sialylation in both F52L and F59L clones, the latter to a greater extent in the SW620 line. Screening for *N*-glycans carrying one or more fucose residues together with α(2,3)-sialylation would allow us to identify the presence of the sLe^x^ antigen [Neu5Ac-α(2,3)-Galβ(1,4)-[Fucα(1,3)]-[NAcGlcβ]-], also known as CD15, whose overexpression is a frequent event in several types of cancers, including CRC. According to our findings, the expression levels of these *N*-glycans were similar in all of the SW480/SW620 clones studied, with no statistically significant differences (Figure 3D), which allows us to infer that changes in core-fucosylation levels would not affect CD15 synthesis.

Regarding the ratio between hexoses (H) and *N*-acetyl-hexosamines (N), three possibilities were envisaged (Figure 3E). The ratio H < N points to the prevalence of *N*-glycans with GalNac (*N*-acetyl-galactosamine residues) in the terminal position, which characterizes the LacdiNAc epitope (GalNAcβ1–4NAcGlcβ1-). Likewise, an H = N ratio corresponds to either a terminal GlcNAc or the presence of a bisecting GlcNAc. On the other hand, an H > N ratio identifies hexose-rich structures, a feature that describes high-mannose or hybrid-type *N*-glycans. Most of the oligosaccharide structures identified belong to the H > N group. In the case of the SW480 line, only the attenuated clone SW480 F52L showed a statistically significant decrease in expression (73.2 ± 1.3% in SW480 NTC vs 64.3 ± 1.3% in SW480 F52L; *p* < 0.05, according to the Student’s *t*-test; Figure 3E). In SW620 cells, the two *FUT8*-attenuated clones showed H > N oligosaccharides more abundantly than their SW620 NTC controls [61.1 ± 1.1% in SW620 NTC vs 68.8 ± 2.6%, (SW620 F52L) and 64.7 ± 3.3% (SW620 F59L); *p* < 0.05 according to the Student’s *t*-test, Figure 3E]. Regarding the H = N group, a clearly different pattern was obtained depending on whether SW480 or SW620 line was considered (Figure 3E). SW480-derived clones showed no statistically significant differences. However, *FUT8*-knockdown SW620 clones displayed significant decreases: 19.8 ± 0.9% (NTC SW620) vs 11.9 ± 1.4% (SW620 F52L) and 13.9 ± 2.3% (SW620 F59L), *p* < 0.01 according to the Student’s *t*-test. Likewise, the H < N group also showed expression changes according to the cell line. Thus, the SW480 cell line registered a statistically significant increase in H < N *N*-glycans in the F52L clone [11.8 ± 0.6% (SW480 NTC) vs 19.4 ± 0.4% (SW480 F52L); *p* < 0.05, Figure 3E] but not in the F59L clone (12.8 ± 1.3%). However, no statistically significant changes in the H < N group were found in SW620-derived clones. In brief, the attenuation in *FUT8* expression coincided in SW480 F52L with an increase in the expression of H < N *N*-glycans and a decrease in H > N oligosaccharide species. In the SW620 line, FUT8 knockdown coincided with a decrease in H = N oligosaccharides in F52L and F59L clones and an increase in H > N *N*-glycans.

Antennarity refers to the number of branches of *N*-glycans depending on the NAcHex-Hex motifs attached to the paucimannosidic trimannosyl-*N*,*N*′-diacetyl-chitobiose. Assuming that this core provides two GlcNAc (N2) and three Man (H3) residues, a diantennary *N*-glycan would have at least one H5N4 sequence, a triantennary *N*-glycan would have at least one H6N5 sequence, a tetra-antennary *N*-glycan would have at least one H7N6 sequence, and a polyantennary *N*-glycan would have at least one H7N6 sequence and above, as schematized in Figure S2. Nevertheless, it should be noted that this classification is not able to discriminate the existence of *N*-glycans bearing LacNAc repeats (−3Gal β1–4GlcNAcβ1-), as in the case of an H6N5 structure that is not a three-branched *N*-glycan, but a diantennary *N*-glycan with three LacNAc motifs. This is the reason we have considered the polyantennary group, which does not refer to *N*-glycans with more than four branches but to sequences that meet the conditions of H > 7 and *N* > 6, which mainly encompass *N*-glycans probably endowed with repeated LacNAc motifs. In other words, they may be with sustained confidence bearing poly[*N*-[*N*-Acetyl-lactosamine]-[di/tri/tetra-]]-antennary structures.

As shown in Figure 3F, of the four *N*-glycan groups classified according to branching and LacNAc repeats, the highest percentage of expression corresponded to polyantennary oligosaccharides in *FUT8*-knockdown SW480 and SW620 clones as well as in wild-type SW480 and SW620 cells. Overall, the clones attenuated for *FUT8* expression showed a statistically significant increase in H5N4 diantennary (*p* < 0.05, according to the Student’s *t*-test) and tri-, tetra-, and polyantennary oligosaccharides (*p* < 0.01, according to the Student’s *t*-test) in the SW480 line (Figure 3F). However, this pattern was inconsistently followed by the SW480 F52L clone since for di- and triantennary groups, no significant changes were observed, while for tetra- and polyantennary *N*-glycans, the magnitude of the increase was not as marked as for the SW480 F59L clone (*p* < 0.05 vs *p* < 0.01, respectively, according to the Student’s *t*-test; Figure 3F). In the SW620 line, the two clones attenuated for *FUT8* showed a substantial increase in expression (*p* < 0.01, Figure 3F).

### Quantitative Analysis by SILAC Tagging and NanoLC-LTQ Orbitrap MS Analysis of the Proteome of the SW480/SW620 sh*FUT8* CRC Cell Model

**Figure 4:**
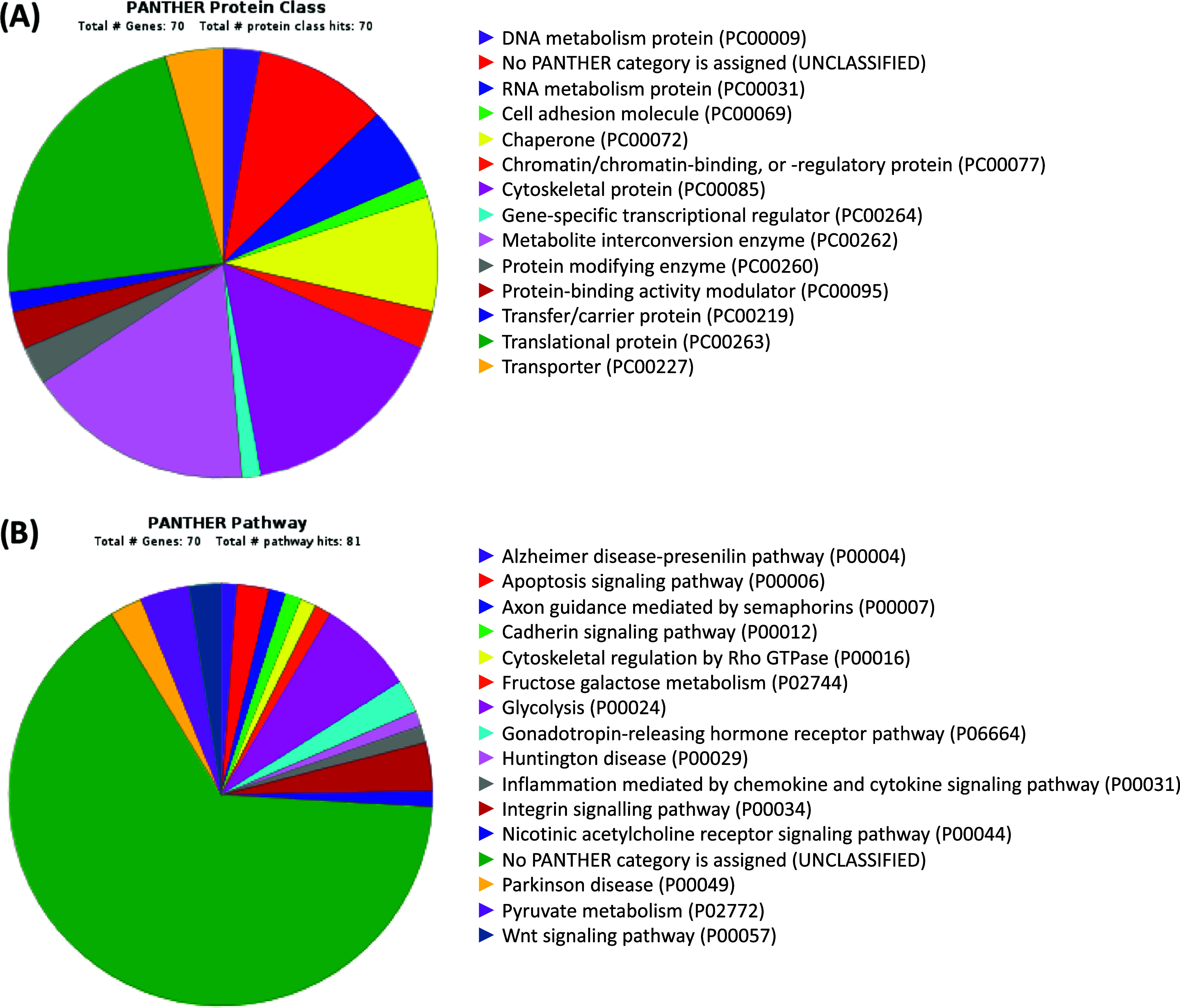
Distribution using PANTHER software of proteins with highly differential expression according to the protein class (A) and the pathways in which they participate (B). PANTHER: Protein Analysis Through Evolutionary Relationships.

On average, the samples showed an isotopic incorporation rate of 0.84 (0.74 for arginine peptides and 0.84 for lysine peptides; Figure S4). Peptide fragmentation was performed using CID and HCD strategies. The software platform MaxQuant’s PTQXC v.0.80.14 confirmed that the quality was equivalent in both modes, but HCD was selected because it provided a greater number of identified proteins. In this regard, a total of 963 proteins were identified from 2 or more unique peptides (Supporting Information 2), of which 190 proteins could be quantified using the log2 fold-change of the normalized values corrected for the conversion from arginine to proline (Table S1). In order to interpret the global changes in the proteome associated with the knockdown of the *FUT8* gene, we selected signals showing the greatest variation (i.e., log 2 fold-change >1 or <1) (Table 2). PANTHER software revealed that the majority of proteins affected in their expression were assigned to RNA metabolism, cell adhesion, cytoskeletal architecture, metabolite enzymes, and translation (Figure 4A). The main cellular pathways affected included fructose–galactose metabolism, glycolysis, pyruvate metabolism, the gonadotropin-releasing hormone receptor pathway, the integrin and cadherin signaling pathways, as well as the Wnt signaling pathway (Figure 4B).

**Table 2:**
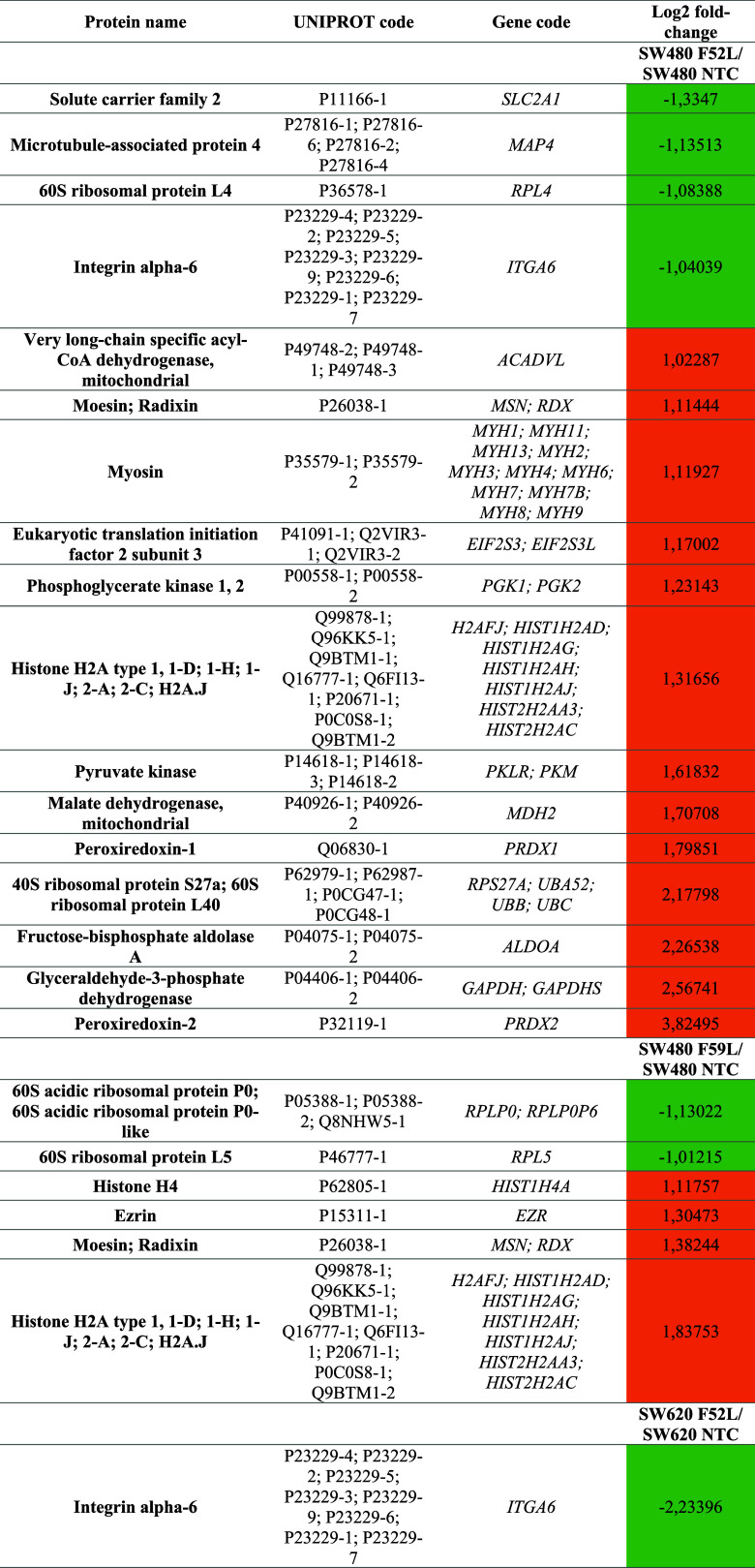
Selected High Differentially Expressed Proteins Detected in the SW480/SW620 shFUT8-Knockdown CRC Cell Model by LC-ESI-LTQ Orbitrap MS after SILAC Tagging^a^

a NTC clones were used as a reference for quantification using the log 2 fold-change method.

**Figure 5:**
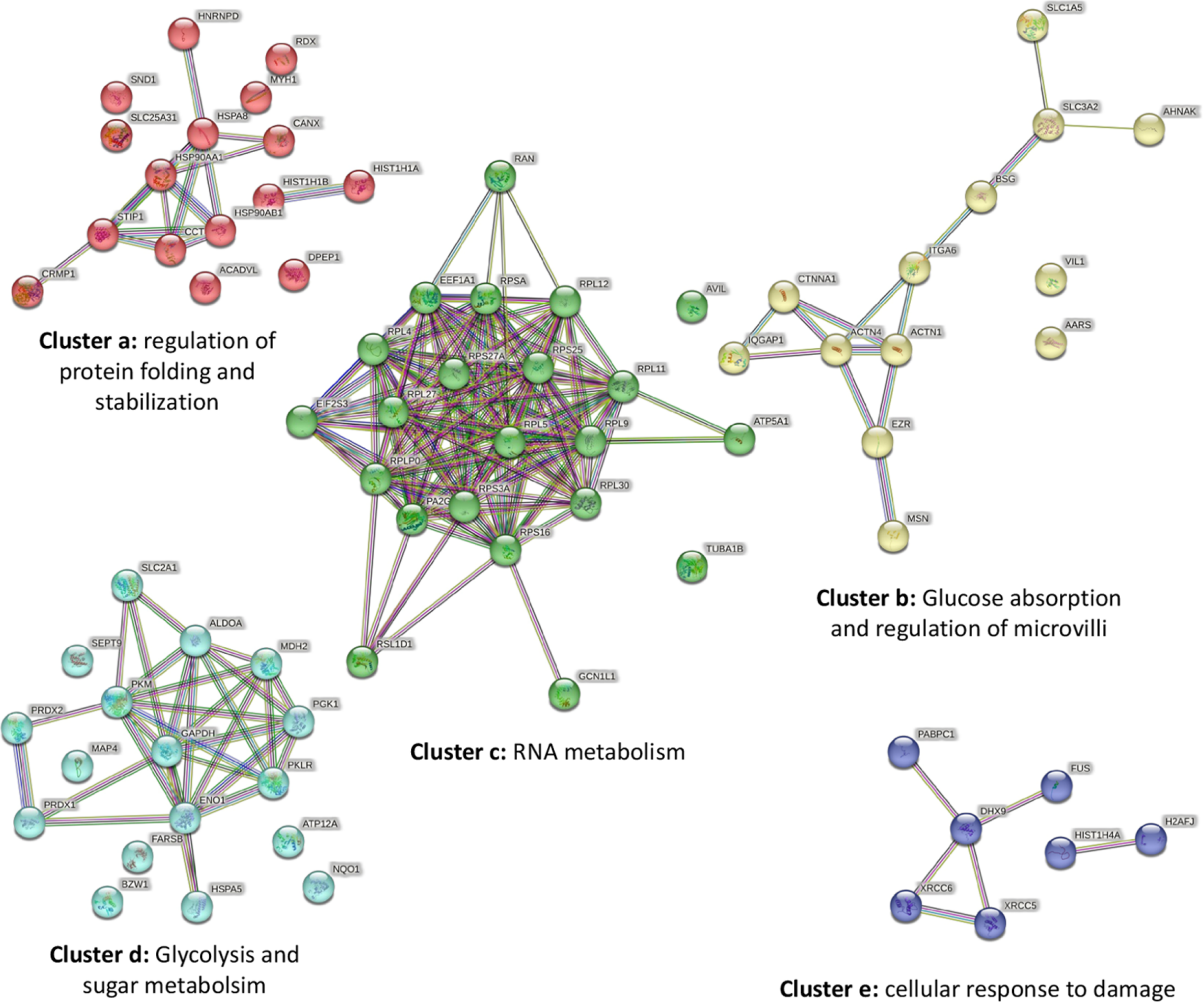
STRING protein–protein interaction network map clusters of the 70 differentially expressed genes in the SW480/SW620 shFUT8 cell model of colorectal cancer. STRING: Protein–protein Interaction Networks Functional Enrichment Analysis.

Subsequently, using the STRING database, the predicted protein–protein interactions were grouped into 5 functional clusters (Figure 5)

(a) Regulation of protein folding and stabilization: ACADVL, CANX, CCT4, CRMP1, DPEP1, HIST1H1A, HIST1H1B, HNRNPD, HSP90AA1, HSP90AB1, HSPA8, MYH1, RDX, SLC25A31, SND1, and STIP1.
(b) Glucose absorption and regulation of microvillus: AARS, ACTN1, ACTN4, AHNAK, BSG, CTNNA1, EZR, IQGAP1, ITGA6, MSN, SLC1A5, SLC3A2, and VIL1.
(c) RNA metabolism: ATP5A1, AVIL, EEF1A1, EIF2S3, GCN1L1, PA2G4, RAN, RPL11, RPL12, RPL27, RPL30, RPL4, RPL5, RPL9, RPLP0, RPS16, RPS25, RPS27A, RPS3A, RPSA, RSL1D1, and TUBA1B.
(d) Glycolysis and sugar metabolism: ALDOA, ATP12A, BZW1, ENO1, FARSB, GAPDH, HSPA5, MAP4, MDH2, NQO1, PGK1, PKLR, PKM, PRDX1, PRDX2, SEPT9, and SLC2A1.
(e) Cellular response to damage: DHX9, FUS, H2AFJ, HIST1H4A, PABPC1, XRCC5, and XRCC6.

## Discussion

Aberrant glycosylation is ever-present in oncogenic transformation, with a capital influence on tumor progression, immune evasion, invasiveness, metastatic potential, and (radio)chemoresistance.^38−41^ Consequently, numerous studies have addressed the importance of glycosylation in several types of cancer, including CRC.^42,43^ However, the impact of aberrant glycosylation is complex because it encompasses the glycosylation pattern and the proteome composition, not exclusively the glycoenzymes.^44^ Furthermore, changes in the glycosylation machinery affect the biological activity and interactome of glycoproteins, magnifying the map of alterations due to impaired glycosylation. This critical consideration has not always been addressed with the degree of attention it deserves,^45,46^ so our study has leveraged the multiomic proteomic and glycomic approach to assess more closely the extent of glycosylation changes in CRC.

Therefore, we integrated label-free semiquantitative profiling of *N*-glycome and protein labeling with SILAC to study the changes in *N*-glycan and protein expression at two stages of CRC progression under the effect of *FUT8* knockdown. *N*-Glycome profiling was performed by *N*-glycan adsorption on PVDF membranes and enzymatic release in combination with chemical derivatization of sialic acids, achieving a high-throughput MALDI-TOF MS analytical methodology using a minute amount of cell extract. Proteomic analysis of the same set of cells combined SILAC labeling with a protein enrichment step and subsequent characterization and quantification of the proteome by LC-ESI-LTQ Orbitrap MS. This approach led us to investigate the *N*-glycome and proteome in cell clones derived from the lentiviral knockdown of the *FUT8* gene in CRC lines SW480 and SW620:^28^ SW480 F52L, SW480 F59L, and SW480 NTC, as well as SW620 F52L, SW620 F59L, and SW620 NTC, in addition to the wild-type SW480 and SW620 cells. These eight tumor lines integrated the experimental system to study in vitro the influence of core fucosylation on CRC malignization since the two distinctive malignant stages of the SW480/SW620 tandem are an accepted model of CRC progression:^47,48^ the nonmetastatic primary SW480 cells and metastatic SW620 cells from a lymph node of the same patient.^47,48^ Indeed, in previous research by our team, we verified the degree of inhibition of *FUT8* expression in the attenuated F52L/F59L clones as well as characterizing their functional phenotype.^28,29^

This study confirmed the type of distribution of complex, hybrid, and high-mannose *N*-glycans (Figure 3A), as described in previous reports.^42,45,49,50^ The glycomic results were also consistent with the picture provided by studies of colorectal tissue and CRC cell lines.^51−53^ Specifically, we identified that the *N*-glycans of the SW480/SW620 lines are mainly complex, followed by high-mannose *N*-glycans and hybrid-type *N*-glycans, as well as a residual percentage of paucimannosidic *N*-glycans. Some studies have reported that high-mannose *N*-glycans may increase by up to 50% in the early stages of CRC.^54^ The divergence with our findings could be explained by the different assignment criteria, in our case, based on the sum of the relative intensities of the different *m*/*z* peaks to improve the robustness of the data analysis. However, the most important question to be resolved is the biological significance of the changes in the *N*-glycome as a consequence of *FUT8* silencing. In this sense, the relative abundance of high-mannose structures could suggest probable incomplete processing of *N*-glycans by the glycosylation machinery because of FUT8 knockdown.^54,55^ Thus, the signal at *m*/*z* = 2067.721 corresponding to the fucosylation of the distinctive *N*-glycan Man9HexNAc2Glc1 (Figure 1A), which tags glycoproteins during folding and quality control in the ER,^56^ is a modification that does not exist in normal processing and may be linked to synthesis defects. It is worth noting that high-mannose, fucosylated *N*-glycans have been proposed as markers of metastasis in breast cancer.^57^ It is a matter of time before more research sheds light on whether this situation is also common in CRC because the increase in high-mannose *N*-glycans has been described as an early predictor of colorectal tumors.^54^ On the contrary, in our CRC model, the inhibition of *FUT8* led to a significant reduction in high-mannose *N*-glycans in favor of the complex-type *N*-glycan structures. It is during subsequent maturation in the Golgi that hybrid and branched *N*-glycans can be converted into complex oligosaccharides.^58^ In this regard, Hakomori and Kannagi postulated the hypothesis of incomplete synthesis and neosynthesis of glycosylated epitopes, initially for sphingolipids but later extended to *N*-glycans.^59−61^ Incomplete synthesis, characterized by the presence of truncated glycan structures, is believed to occur in the early stages of the tumor process due to damage to the normal synthesis chain.^60,61^ Neosynthesis is believed to be associated with advanced stages because of the induction of genes involved in the *de novo* synthesis of antigen determinants.^60,61^ These new modalities of advanced *N*-glycan processing can be reviewed in three events: (I) the addition of monosaccharides to the core, such as the core fucose or bisecting GlcNAc; (II) the elongation and (III) maturation of the antennas. Compared with normal epithelium, CRC cells have elevated levels of high-mannose *N*-glycans.^62^ Several *N*-glycome studies have demonstrated the differential distribution of glycan species depending on the stage of CRC.^63,64^ If we stick to the hypothesis of incomplete synthesis and/or neosynthesis, the silencing of *FUT8* in the SW480 and SW620 lines would favor neosynthesis, as suggested by the significant expression of complex forms; consequently, their cellular malignancy would be enhanced. However, we cannot fully endorse this sole possibility since the hyperexpression of complex *N*-glycans is also associated with increased antennal decoration and branching. Therefore, we need to investigate other changes in the *N*-glycome of our CRC model, as shown below.

Regarding the degree of sialylation and the expression of CRC-associated epitopes such as sialyl-Lewis antigens, our study found a significant alteration of α(2,6)-sialylation as a consequence of *FUT8* silencing. Cell surface α(2,6)-sialylation has been associated with metastasis and therapeutic failure in CRC.^65^ However, if we look at the evolution of the expression levels of ST6Gal1, the enzyme responsible for α(2,6)-sialic acid binding during CRC progression, the situation is similar to that described for FucT-8 because its levels increase substantially in the early stages and then decrease.^66,67^ Indeed, studies have reported a significantly higher ST6Gal1 expression in nonmetastatic I–II stage CRC lesions than in metastatic III or IV stage lesions.^68^ It is worth noting that, although the role of ST6Gal1 seems to be eminently pro-tumoral, it has also been described as tumor suppressive in some types of cancer by reducing cell invasion, proliferation, or chemoresistance.^67^ Regarding CRC, cells with upregulated ST6Gal1 expression and enhanced α(2,6)-sialylation developed chemoresistance to Cetuximab or Gefitinib, both affecting EGFR.^69,70^ As we have found that silencing the *FUT8* gene is associated with increased α(2,6)-sialylation, a negative response to these drugs would be expected, and thus, we have recently described the cellular response to Cetuximab after *FUT8* attenuation.^29^ On the other hand, it has been reported that upregulated ST6Gal1 may also reduce the migration capacity of cancer cells due to the impairment of intercellular adhesion molecule-1 (ICAM-1), and vice versa when α(2,6)-sialylation is attenuated.^71^ In correspondence with the increased levels of α(2,6)-sialylation in the *FUT8*-attenuated clones of the SW480 and SW620 lines (Figure 3C), the results of functional assays indicated a reduced migration capacity in our SW480 *FUT8*-knockdown cells.^28^

As for the likely presence of bisecting GlcNAc residues in our *N*-glycome results, a significant reduction in oligosaccharide structures fulfilling the Hex = HexNAc relationship was shown in the *FUT8*-attenuated clones of the SW620 line. A close relationship has been previously described between the function of FucT-8 and *N*-acetylglucosaminyltransferase III (GlcNAc-T III) since both act directly on the *N*-glycan core.^72^ More specifically, the presence of a bisecting GlcNAc hinders the downstream activity of FucT-8,^73^ so both modifications introduce notable conformational constraints to the glycosidic chain, although those of bisecting GlcNAc are substantially greater.^74^ In *FUT8*(−/−) mouse embryonic fibroblasts, Kurimoto and co-workers found that the absence of core fucosylation was associated with an increased expression of GlcNAc-T III and the presence of bisecting GlcNAc oligosaccharides.^75^ To our knowledge, no further extensive investigations have been conducted on this topic, as other groups attempted to assess the changes linking carbohydrate chain structure to bisecting GlcNAc and *FUT8*-associated regulation.^76^ However, several promising investigations on the membrane proteins of different CRC cell lines have demonstrated that metastatic LIM1215 or SW620 cells can exhibit high levels of bisecting GlcNAc *N*-glycans as compared with nonmetastatic cancer cell lines.^42,45,77^ Interestingly, the proportion of bisecting GlcNAc and core-fucosylated *N*-glycans appeared to be correlated during CRC progression, although their expression varied greatly and did not follow linear trends.^77^ Thus, bisecting GlcNAc was strongly expressed in the metastatic line LIM1215, comparatively less so in the moderately differentiated primary line LIM1899, and was absent in the poorly differentiated line LIM2405.^77^ In contrast, core fucosylation was high in the primary LIM1899 CRC cell lines, lower in the metastatic LIM1215 cells, while significantly higher in the aggressive poorly differentiated LIM2405 cells.^77^ In our case, the knockdown of the *FUT8* gene did not appear to be associated with an immediately increasing level of bisecting structures in the attenuated clones of the SW480/SW620 lines, although the authors acknowledge that it would be necessary to differentiate exhaustively the structures that comply with the Hex = HexNAc relationship carrying a bisecting GlcNAc from the truncated forms. Nevertheless, our results reinforced the hypothetical antagonistic relationship between bisecting GlcNAc and core-fucosylated glycoforms, as well as its multidimensional nature being more influenced by the tumor stage than if it were a mere mechanistic coupling. Furthermore, the literature has often described the presence of bisecting GlcNAc as a suppressing metastasis marker.^78^ Similarly, the high expression of bisecting GlcNAc has been reported in the metastatic SW620 line as well as in the well-differentiated I-stage SW1116 line, in contrast to very low levels in the nonmetastatic SW480 line.^45^ Accordingly, other glycosylated structures fulfilling the Hex = HexNAc relationship are higher in SW480 than in SW620 cells (Figure 3D). These observations confirm the need for further in-depth studies on CRC evolution to address the expression and catalytic potential of glycosylation enzymes, as well as the MS/MS elucidation of their oligosaccharide products to resolve the apparent discrepancies between the glycosylation machinery that remains active and the resulting cellular glycophenotype.

Enhanced fucosylation is a common event reported in earlier stages of CRC, which progressively decreases until later stages of cancer progression.^27,79^ To our knowledge, few or almost no studies have been performed on the adaptation of fucosylation enzymes to the loss of the FucT-8 enzyme. A recent publication reporting *N*-glycome alterations in the murine colon adenocarcinoma cell line MC38 after fully restoring FucT-4 and FucT-9 activity^80^ reported the neosynthesis of Le^x^ antigens along with modification of core fucosylation, sialylation, and antennarity, which agrees with our findings showing enhanced multifucosylation concomitant with reduced monofucosylation in *FUT8*-knockdown clones (Figure 3B). AAL blotting in FucT-4/FucT-9 revertant CRC cells was lower than in the mock line MC38, in which only FucT-8 was active, consistent with our observation that reducing FucT-8 expression results in increased multifucosylation by other different glycosyltransferases.

Changes in antennarity were among the most evident observed in the *N*-glycan profiling of our CRC model, e.g., the increased antennarity in the *FUT8*-attenuated cells (Figure 3E). It should be noted, as indicated in the calculation method of *N*-glycan traits (Table 1), that the presence of poly-LacNAc can only be evaluated with certainty in those structures that meet the ratios ∑[(Hex) > 7] and (HexNAc) > 6. Upregulated *N*-glycan antennarity has also been described in the tumor tissue of CRC patients in comparison with control tissue,^81^ as well as the presence of highly branched *N*-glycans in stage II CRC cells compared to normal epithelial cells.^62^ Regarding antennarity, it has been recently reported that FucT-8 activity is largely determined by the peptide sequence of the protein backbone in paucimannosidic and high-mannose *N*-glycans.^82^ However, for complex *N*-glycans, FucT-8 activity is essentially modulated by the presence of glycosites, with a preference for the diantennary and triantennary *N*-glycans generated by GnT-4 but not for the triantennary *N*-glycans generated by GnT-5 or for tetra-antennary *N*-glycans.^82−85^ In this regard, in examining the correlation of core fucosylation with the branching of *N*-glycans (Figure 3E), we found that the reduced expression of FucT-8 in SW480/SW620 *FUT8*-knockdown cells was significatively associated with a higher degree of branching, especially for tetra-antennary *N*-glycans. As it has been postulated that FucT-8 prefers complex *N*-glycans rather than low-/high-mannose *N*-glycans,^82^ we believe that it would be interesting to explore in the future the possible mechanisms that explain how the reduction of FucT-8 is connected to the magnification of antennarity in CRC.

Another aspect to highlight is the correlation between polylactosamine levels and enhanced invasion and metastasis, as seen in SW620, LS174T, and LoVo CRC cells.^86^ In a previous study, we reported increased proliferation, colony formation, and a more mesenchymal phenotype in *FUT8-*knockdown SW480 cells,^28^ which is consistent with the current findings of highly branched/polylactosamine *N*-glycans in SW480 cells defective in *FUT8* expression. Contrarily, *FUT8*-attenuated SW620 cells appear to exceed the regulatory capabilities of FucT-8, as previously suggested^28,29^ since the currently observed modifications in the antenna profile did not worsen their natural metastatic behavior. However, we must remember that the concomitant presence of polylactosamine with multifucosylation has been associated with a less invasive and less aggressive phenotype in some CRC cells in early malignant stages,^42,87,88^ which could explain why, despite overexpressing mesenchymal markers, the clones of the SW480 line silenced for *FUT8* were not necessarily more aggressive.^28^ Although these are results to be taken into consideration, the selection criteria in the case of di-, tri-, and tetra-antennary groups are very strict, and by working solely with the number of hexoses and *N*-acetyl-hexosamines, no chain lengthening, such as sialylation or fucosylation, was contemplated. In summary, the attenuation of *FUT8* expression in the SW480 and SW620 lines coincided with a general increase in *N*-glycan branching, especially with the presence of polylactosamine.

The SW480/SW620 sh*FUT8* CRC model has shown that *FUT8* silencing has direct consequences on oligosaccharide chains and also on the expression of protein species *a priori* not affected by fucosylation or that do not participate in the regulation of glycosylation. More specifically, we found that the affectation of core fucosylation produces changes that are not restricted to the membrane proteome but extend to a broad array of protein species encompassed in 5 clusters according to their functional profile (Figure 5). Cluster a included proteins involved in the regulation of protein folding and stabilization. The failure of protein architecture can be traced back to the ER-Golgi axis, which is responsible for the synthesis, maturation, folding, and trafficking/export of cellular proteins.^89^ ER stress caused by abnormal accumulation of unfolded or misfolded (glyco)proteins triggers the unfolded protein response (UPR) to reprogram transcriptional, translational, and post-translational mechanisms.^90^ Interestingly, defects in the expression of glycosyltransferases such as FucT-2 can activate the UPR,^91^ although this is an ER-localized enzyme. Alterations in glycosylation in the Golgi apparatus also elicit UPR-mediated responses.^92^ FucT-8 is in the cis-Golgi and, unlike other glycosyltransferases, has an SH3 domain that is believed to be critically responsible for the cellular localization and catalytic activity of the enzyme.^85,93^ Therefore, it is not unreasonable to assume that FucT-8 malfunction, or aberrant expression, causes ER stress, as our results suggest. On the other hand, chaperones such as heat shock proteins or chaperone lectins may exert their biochemical functions in this situation,^94,95^ as we found after the knockdown of the *FUT8* gene. This is the case of increased levels of calnexin (CANX), which has been described as a potential biomarker of poor prognosis in CRC patients and whose knockdown in HCT116 CRC cells led to increased chemosensitivity to 5-FU and reduced clonogenic survival.^96^ Likewise, heat shock proteins such as HSP90AA1, HSP90AB1, and HSPA8 were also altered in CRC. HSP90AA1 is upregulated in colorectal polyps with a high degree of dysplasia and the potential for becoming malignant.^97^ Similarly, HSP90AB1 is commonly affected in various malignant diseases, and reduced expression in CRC is indicative of poor prognosis.^98,99^ Our proteome screening showed a reverse expression trend for HSP90AB1, as *FUT8*-attenuated clones from the SW480 line overexpressed HSP90AB1, while in their metastatic SW620 counterparts, expression was reduced. HSPA8 belongs to the HSP70 family of 13 members;^100^ their landscape in CRC is complicated, as some of them are overexpressed and others display reduced expression.^101^ HSPA8 specifically is usually overexpressed in tumors, although indicating a favorable outcome.^101,102^

On the other hand, we also identified proteins participating in cell polarity and microvilli (cluster b, Figure 5). Fucosylation, including core fucosylation, has historically been recognized as crucial to cell–cell interaction and downstream signaling.^103^ Therefore, proteins regulating cell polarity could be potential targets of *FUT8* silencing, as we found. α-Actinins (ACTNs) cross-link actin filaments at focal adhesions and regulate cell migration. Overexpression of the isoform actinin-4 (ACTN4) enhanced cancer cell motility, lymph node invasion, and metastasis in DLD-1 and SW480 CRC cells.^104,105^ Similarly, IQGAP1 is a scaffolding protein that participates in cell dynamics and is expressed in microtubules at the cytoplasmic side of the nuclear envelope.^106^ However, overexpression at the invasion front of tumoral spots is frequently observed, and cells are prone to detach, therefore participating in metastasis.^106,107^ Several drugs that affect the functions of IQGAP1 have been assayed.^106,108,109^ Likewise, SLC1A5 is an important transporter of glutamine frequently overexpressed in various cancer cells, including CRC, and affected by chemotherapy.^110−112^

Proteins related to the damage response deserve attention as they may be related to radio- and/or chemoresistance or drug sensitivity, thus encompassing potential drug targets through the inhibition of FucT-8 activity (cluster c, Figure 5). In this sense, it has recently been described that deregulated fucosylation can affect many intracellular proteins, including the ribosomal protein S3, which participates in nuclear DNA repair.^113^ Indeed, the fucosylation and sialylation inhibitor pictilisib affected the DNA repair capacity of lung cancer cells.^114^ The histone H2AFJ (cluster e) is usually expressed in luminal epithelial cells, although its role is still poorly understood.^115^ However, it was recently found that high H2AFJ expression in CRC cells correlated with a significantly worse prognosis and acquired chemoradiation resistance.^116^ Interestingly, this group of proteins also revealed other specimens with an important role in the cellular response to radiotherapy, such as XRCC5 and XRCC6. Together, they form the XRCC5/6 heterodimer that binds to DNA double-strand break ends and triggers the nonhomologous end joining (NHEJ) pathway of DNA repair. Likewise, *XRCC5* silencing led to an enhanced cisplatin radiosensitization in a cervical carcinoma cell line model,^117^ and *XRCC6*-knockdown enhanced radiosensitivity in mammal cells,^118^ and chemosensitivity to cisplatin in bladder cancer.^119^ These results indicate that the inhibition of XRCC5 or XRCC6 improves the effectiveness of chemotherapy used in CRC, such as platinum-based drugs and/or radiotherapy. However, the downregulation of XRCC5 or XRCC6 is also associated with a poorer outcome of CRC;^120,121^ one possible explanation is the intracellular localization of these proteins. In this regard, SW480 and SW620 cells manifest opposite responses to radiotherapy: the former is radioresistant and the latter is radiosensitive.^122^ It is worth noting that SW480 displays greater expression of the XRCC5/6 dimer in the cytosol, while in SW620, cytosolic levels are negligible in contrast to extensive nuclear expression.^122^ Our approach did not allow the cell location of XRCC5/6, although we have seen a striking reduction in the levels of both protein species in the *FUT8*-silenced SW620 F52L clone. This preliminary evidence suggests that metastatic CRC may respond favorably to treatment regimens targeting *FUT8*/FucT-8. Interestingly, we have recently reported a greater sensitivity of SW620 F52L and F59L clones to oxaliplatin, although not statistically significant at the selected dosage.^29^ Consequently, it would be interesting to conduct more detailed future studies on the response of SW480/SW620 cells to (chemo)radiotherapy.

## Conclusions

Cellular models are suitable systems for evaluating changes in the expression of target genes and/or proteins and how they contribute to dyshomeostasis and cancer progress. Additionally, in vitro assays are exceptionally useful for understanding how cancer cells respond to (chemo)radiotherapy in a straightforward way. In this regard, the SW480/SW620 sh*FUT8* CRC cell model has provided novel insights into the understanding of CRC molecularity. Specifically, it allowed us to observe the substantial changes that occur in the *N*-glycome and proteome of the SW480/SW620 cell tandem as an effect of the shRNA-dependent knockdown of the *FUT8* gene. The value of the data collected is that they come from a cellular model that subsumes the complexity of the tumor process by being composed of the primary line SW480 and its isogenic metastatic counterpart SW620. However, the current results leave unanswered questions surrounding the glycomic picture of each stage of CRC and thus achieve a correct and complete vision of its molecular evolution. However, the current results call for more detailed research of the glycomic picture of each stage of CRC to obtain an overview of its molecular evolution. Specifically, the extensive *N*-glycome microheterogeneity in our cellular model needs to be examined in detail in order to understand more clearly the impact that core fucosylation has on the expression and/or activity of protein mediators that drive CRC.

## Supporting Information Available

SIMCA and PCA analysis for glycomic data from the SW480/SW620 shFUT8 cell model (Figure S1); representative MS2 fragmentation pattern of a monofucosylated *N*-glycan, and a α(2,6)-terminal sialic acid *N*-glycan (Figure S2); *N*-glycosidic structures assigned to *m*/*z* signals under the classification criteria of Glyco-Peakfinder software (Figure S3); graph showing the average isotopic incorporation rate in SILAC experiments (Figure S4); and list of differentially expressed proteins detected in the SW480/SW620 shFUT8 cell model by LC-ESI-LTQ Orbitrap MS after SILAC (Table S1) (PDF)
All *m*/*z* data used for the *N*-glycomics analysis of the SW480/SW620 shFUT8 cell model (XLSX)
All *m*/*z* data used for the proteomics analysis of the SW480/SW620 shFUT8 cell model (XLSX)

## Notes

Alterations in the glycomic profile are capable of modulating oncogenesis, as well as progression, therapeutic response, and metastasis. Specifically, increased core fucosylation is one of the key events in the early stages of colorectal cancer (CRC). We report that decreasing the degree of core fucosylation by knockdown of the FUT8 gene clearly affects the distribution of glycan traits such as *N*-glycan type, glycan epitopes, and antennarity. In addition, the repression of FUT8 also affects the expression of proteins involved in endoplasmic reticulum homeostasis and stress response or cell polarity, thus helping to modulate cell phenotype and behavior.

## Abbreviations

5-FU: 5-fluorouracil
AmBic: ammonium bicarbonate
ACN: acetonitrile
ALL: *Aleuria aurantia* lectin
AmBic: ammonium bicarbonate
CID: collision-induced dissociation
CIMP: CpG island methylator phenotype
CIN: chromosome instability
DHB: 2,5-dihydroxybenzoic acid
DMEM: Dulbecco’s modified Eagle’s medium-high glucose
DOC: sodium deoxycholate detergent
DTT: dithiothreitol
EDC: 1-ethyl-3-(3-(dimethylamino)propyl)carbodiimide
ER: endoplasmic reticulum
FA: formic acid
FBS: fetal bovine serum
GuHCl: guanidinium chloride
FOBT: fecal occult blood
ESI-LTQ Orbitrap MS: liquid chromatography-electrospray ionization-linear trap Orbitrap mass spectrometry
LCA: *Lens culinaris* agglutinin
MALDI-TOF MS: matrix-assisted laser desorption/ionization-time of flight mass spectrometry
MSI: microsatellite instability
*m*/*z*: mass-to-charge ratio
NHEJ: nonhomologous end joining
NP-40: Nonidet P-40 detergent
NTC: non-targeted control
PANTHER: protein analysis trough evolutionary relationships
PCA: principal component analysis
PNGase F: peptide: *N*-glycosidase F
PVDF: poly(vinylidene difluoride)
SDS: sodium dodecyl sulfate
shRNAi: short hairpin RNA interference
SILAC: stable isotopic labeling of amino acids in cell culture
SIMCA: soft independent modeling by class analogy
STRING: protein–protein interaction networks functional enrichment analysis
TFA: trifluoroacetic acid
UPR: unfolded protein response
