# Supplementary material for: High-Throughput Mass Spectrometry Analysis of N-Glycans and Protein Markers after FUT8 Knockdown in the Syngeneic SW480/SW620 Colorectal Cancer Cell Model
Source: J Proteome Res. 2024 Mar 20;23(4):1379–98. doi: 10.1021/acs.jproteome.3c00833 (PMC11002942; doi:10.1021/acs.jproteome.3c00833)
Supplement: Supplementary file 1 — pr3c00833_si_001.pdf [file pr3c00833_si_001.pdf]

Supporting information

High-throughput mass spectrometry analysis of N-glycans and protein markers after *FUT8* knockdown in the syngeneic SW480/SW620 colorectal cancer cell model

Rubén López-Cortés[1]; Laura Muinelo-Romay[2]; Almudena Fernández-Briera[3#]; Emilio Gil Martín[4#*]

[1] Doctoral Program in Methods and Applications in Life Sciences, Faculty of Biology, Universidade de Vigo. Campus Lagoas-Marcosende, 36310, Vigo, Pontevedra (Galicia), Spain. rlcortes.eu@gmail.com

[2] Liquid Biopsy Analysis Unit, Translational Medical Oncology (Oncomet), Health Research Institute of Santiago de Compostela (IDIS), CIBERONC. Travesía da Choupana, 15706, Santiago de Compostela, A Coruña (Galicia), Spain. lmuirom@gmail.com

[3] Molecular Biomarkers, Biomedical Research Centre (CINBIO), Universidade de Vigo. Campus Lagoas-Marcosende, 36310, Vigo, Pontevedra (Galicia), Spain. abriera.fernandez@gmail.com

[4] Nutrition and Food Science Group, Department of Biochemistry, Genetics and Immunology, Faculty of Biology, Universidade de Vigo. Campus Lagoas-Marcosende, 36310, Vigo, Pontevedra (Galicia), Spain. egil@uvigo.es

[#] These authors contributed equally to this work.

[*] Corresponding author: Emilio Gil Martín; phone: +34 (986) 812 570; email: egil@uvigo.es

Table of content

Figure S1: SIMCA and PCA analysis for glycomic data from the SW480/SW620 shFUT8 cell model......3

Figure S2: Representative MS2 fragmentation pattern of (A) a monofucosylated N-glycan, and (B) a α(2,6)-terminal sialic acid N-glycan..4

Figure S3: N-glycosidic structures assigned to m/z signals under the classification criteria of the Glyco-Peakfinder software...5

Figure S4: Graph showing the average isotopic incorporation rate in SILAC experiments.........................6

Table S1: list of differentially expressed proteins detected in SW480/SW620 shFUT8 cell model by LC-ESI-LTQ Orbitrap MS after SILAC tagging..7

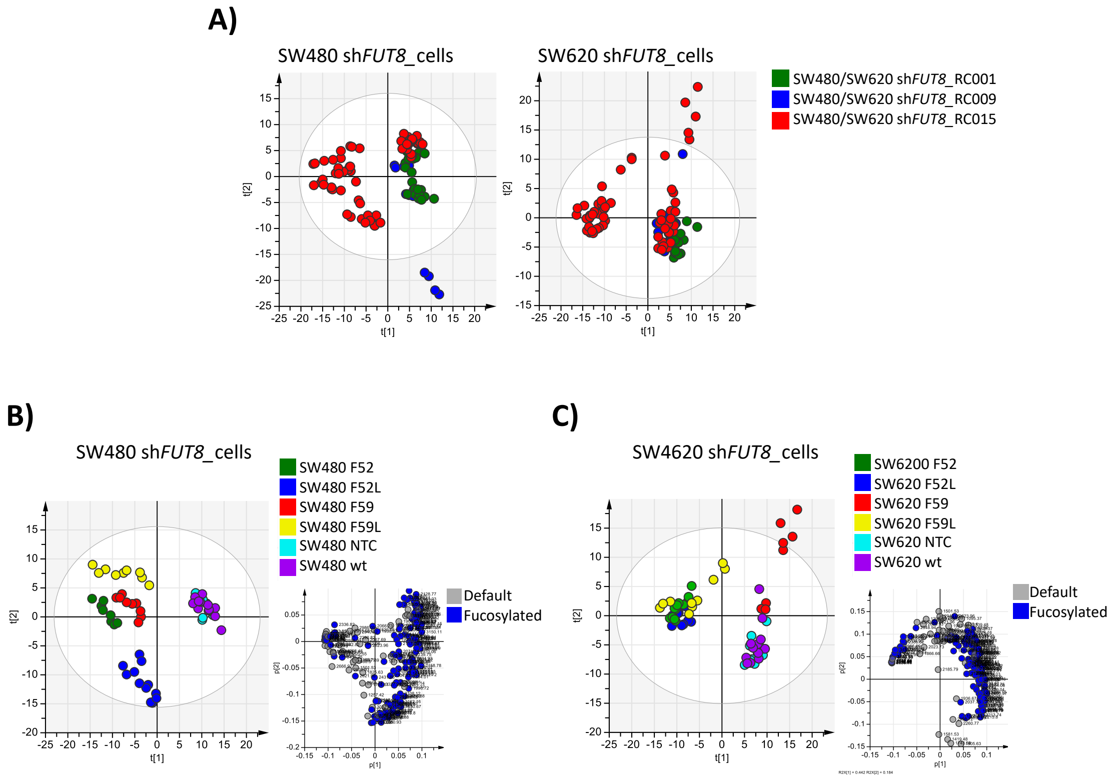

Figure S1: (**A**) SIMCA analysis to identify a potential batch-effect between replicates. Each replicate was distinctively coloured (green, RC001; blue RC009; red, RC015), with each dot corresponding to a spot printed onto the MALDI plate. One technical experiment consisted of three biological replicates per glycan and the cell lines were unit-variance scaled and used for multivariate data analysis in SIMCA V13 (Umetrics AB). (**B**, **C**) PCA score plots for SW480 (B) and SW620 (C) of PC1 (40.4% and 44.2%) against PC2 (both 18.4%) coloured according to cell line; (**C**, **D**) Loading plots of PCA model displaying PC1 vs. PC2 coloured in blue according to the presence of fucose in the N-glycan structure. SIMCA: Soft Independent Modelling by Class Analogy. PCA: Principal Component Analysis.

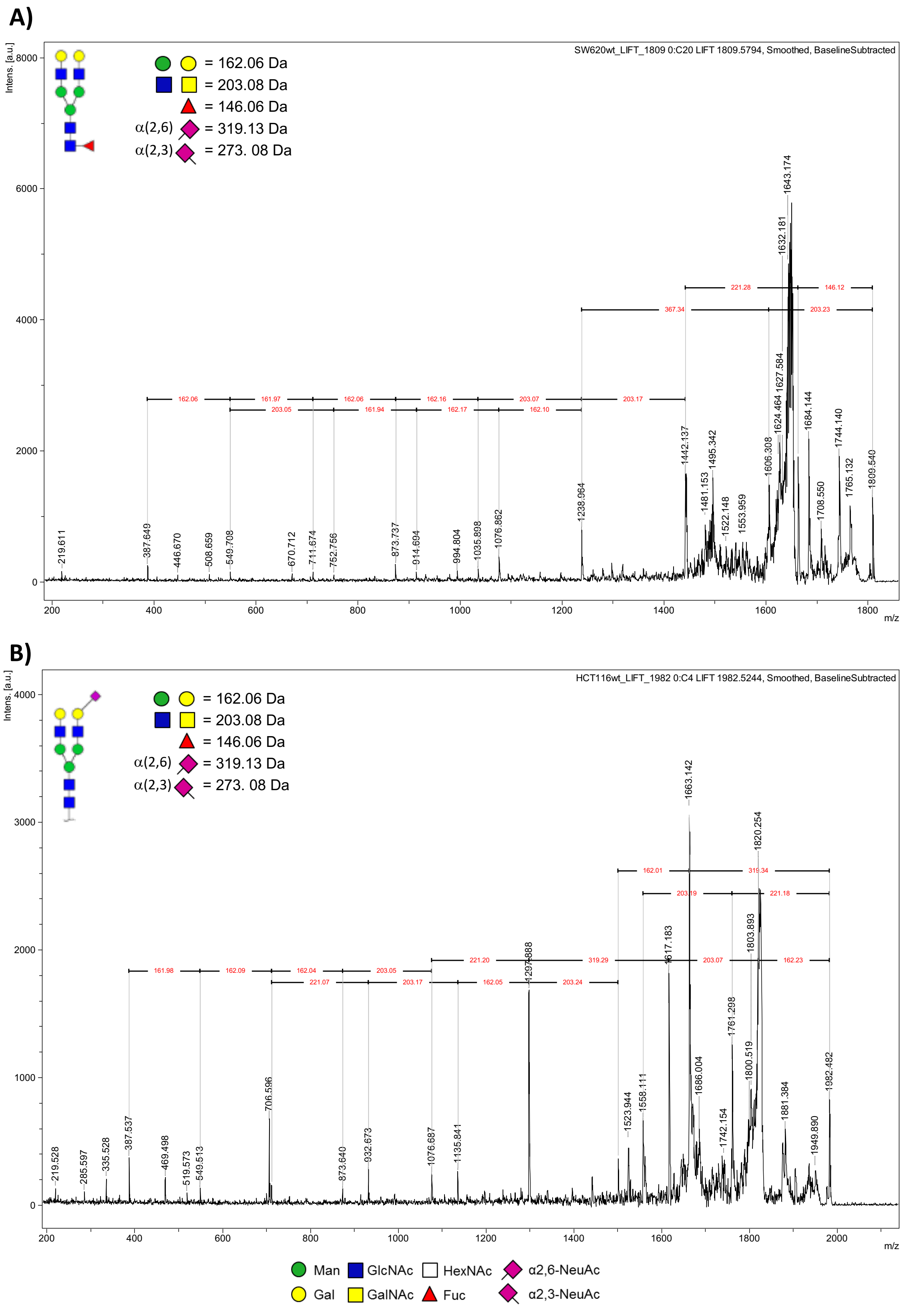

Figure S2: (**A**) Representative fragmentation pattern of a monofucosylated glycan, which can be identified by the loss of 146.06 Da of m/z. (**B**) Representative fragmentation pattern of a glycan with an α(2,6)-terminal sialic acid, which can be identified by the loss of 319.13 Da of m/z due to the chemical derivatization step. Annotation was performed using GlycoWorkbench 2.1 and the SNFG notation: Symbol Nomenclature For Glycans (SNFG) – NCBI. Available at: https://www.ncbi.nlm.nih.gov/glycans/snfg.html.

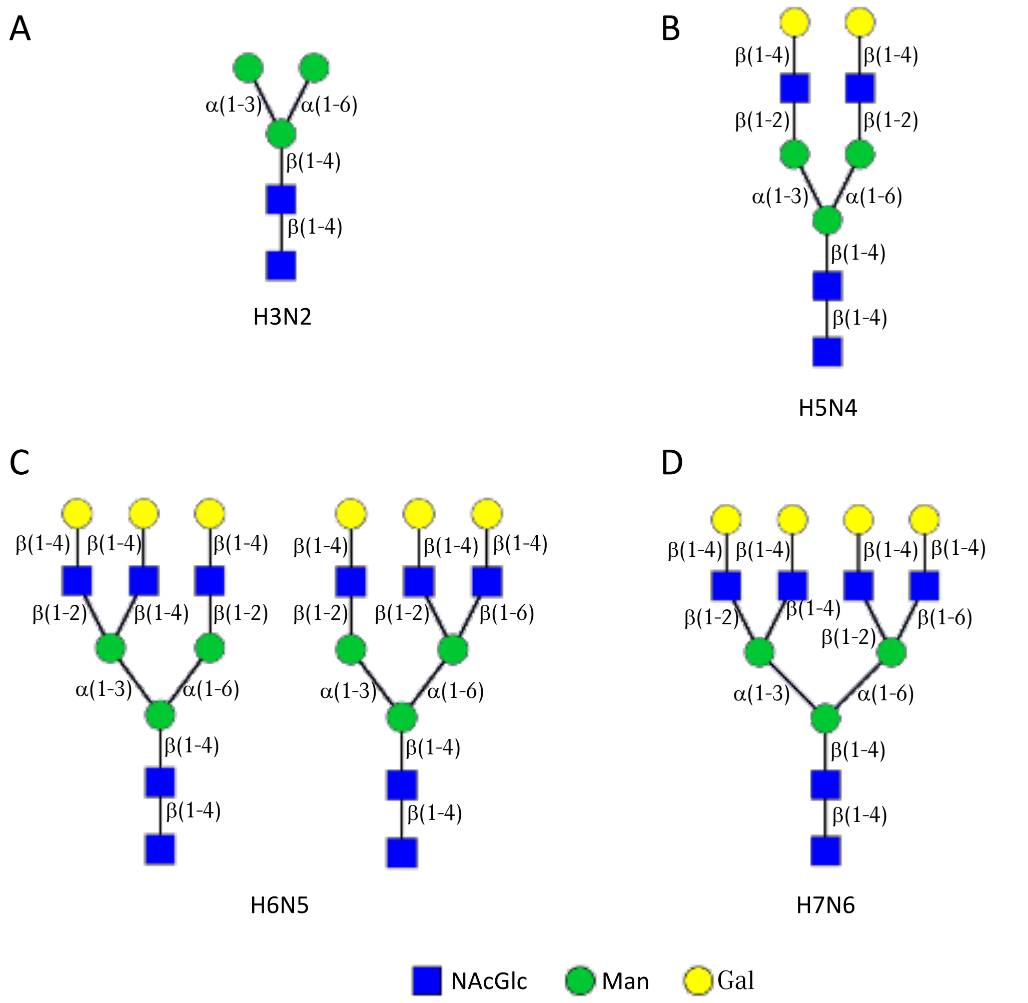

Figure S3: N-glycosidic structures assigned to m/z signals under the classification criteria of the Glyco-Peakfinder software. (**A**) Basic paucimannosidic structure H3N2. (**B**) Di-antennary structure H5N4. (**C**) Tri-antennary structure H6N5. (**D**) Tetra-antennary structure H7N6. The SNFG notation was used: Symbol Nomenclature for Glycans (SNFG) – NCBI. Available at: https://www.ncbi.nlm.nih.gov/glycans/snfg.html.

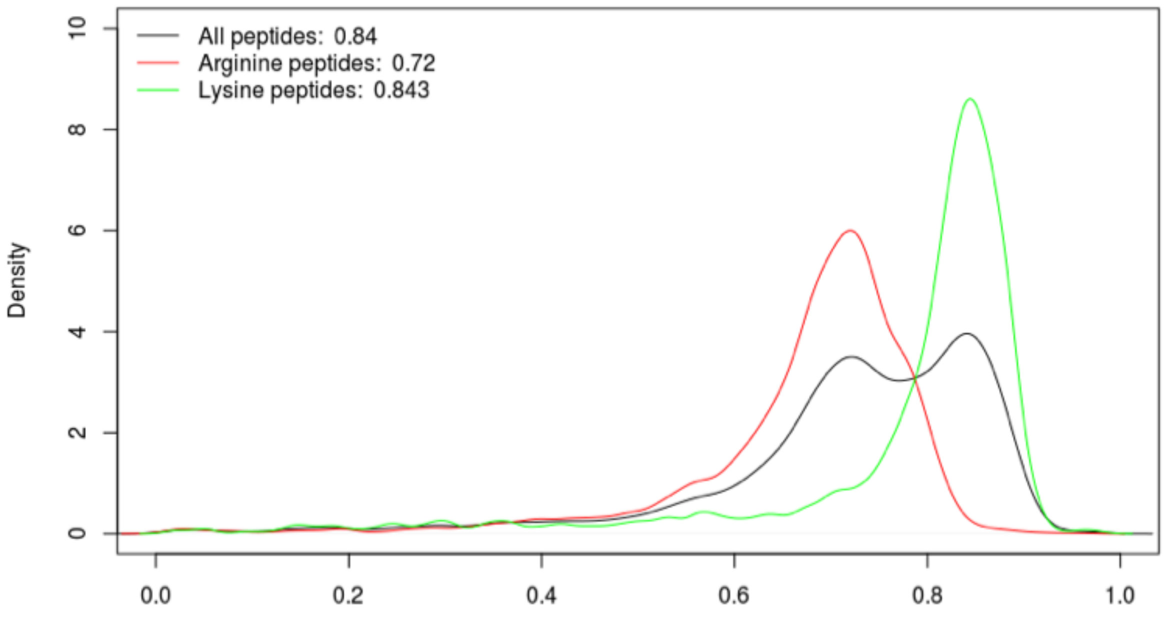

Figure S4: Graph showing the average isotopic incorporation rate for the set of cell samples maintained in a labelled medium for the SILAC experiment. Black line: set of all peptides; red line: peptides with arginine; green line: peptides with lysine.

Table S1: Specific differentially expressed proteins detected in SW480/SW620 sh*FUT8* cell model by LC-ESI-LTQ Orbitrap MS after SILAC tagging. NTC clones was used as reference for quantification using the log2 fold-change method.

UNIPROT code	Protein name	Gene code	SW480 F52L/ SW480 NTC
**P11166-1**	Solute carrier family 2	*SLC2A1*	-1,3347
**P27816-1; P27816-6; P27816-2; P27816-4**	Microtubule-associated protein 4	*MAP4*	-1,13513
**P36578-1**	60S ribosomal protein L4	*RPL4*	-1,08388
**P23229-4; P23229-2; P23229-5; P23229-3; P23229-9; P23229-6; P23229-1; P23229-7**	Integrin alpha-6	*ITGA6*	-1,04039
**Q09666-1**	AHNAK; AHNAK2	*AHNAK; AHNAK2*	-0,973989
**Q9UQ80-1; Q9UQ80-2**	Proliferation-associated protein 2G4	*PA2G4*	-0,864032
**P62241-1**	40S ribosomal protein S8	*RPS8*	-0,832482
**P62753-1**	40S ribosomal protein S6	*RPS6*	-0,775103
**Q15019-1; Q15019-3; Q15019-2**	Septin-2	*SEPT2*	-0,745034
**Q9UHD8-7; Q9UHD8-2; Q9UHD8-5; Q9UHD8-1; Q9UHD8-3; Q9UHD8-4; Q9UHD8-9; Q9UHD8-8**	Septin-9	*SEPT9*	-0,730455
**P12956-1; P12956-2**	X-ray repair cross-complementing protein 6	*XRCC6*	-0,729405
**P30050-1; P30050-2**	60S ribosomal protein L12	*RPL12*	-0,699709
**P05388-1; P05388-2; Q8NHW5-1**	60S acidic ribosomal protein P0	*RPLP0; RPLP0P6*	-0,697817
**P08195-2; P08195-3; P08195-1; P08195-4**	4F2 cell-surface antigen heavy chain	*SLC3A2*	-0,62205
**Q13283-1**	Ras GTPase-activating protein-binding protein 1	*G3BP1*	-0,546242
**Q02878-1**	60S ribosomal protein L6	*RPL6*	-0,505495
**P16401-1**	Histone H1.5	*HIST1H1B*	-0,482678
**P26599-1; P26599-2; P26599-3**	Polypyrimidine tract-binding protein 1; Polypyrimidine tract-binding protein 3	*PTBP1; PTBP3*	-0,481128
**P35221-1; P35221-2; P35221-3**	Catenin alpha-1; Catenin alpha-3	*CTNNA1; CTNNA3*	-0,479321
**O60841-1**	Eukaryotic translation initiation factor 5B	*EIF5B*	-0,435483
**Q07020-1; Q07020-2**	60S ribosomal protein L18	*RPL18*	-0,356521
**P52272-2; P52272-1**	Heterogeneous nuclear ribonucleoprotein M	*HNRNPM*	-0,318915
**P46777-1**	60S ribosomal protein L5	*RPL5*	-0,306047
**Q7KZF4-1**	Staphylococcal nuclease domain-containing protein 1	*SND1*	-0,239959
**Q9ULV4-1; Q9ULV4-2; Q9ULV4-3**	Coronin-1C	*CORO1C*	-0,191633
**P06748-2; P06748-1; P06748-3**	Nucleophosmin	*NPM1*	-0,190934
**P84098-1**	60S ribosomal protein L19	*RPL19*	-0,152604
**P62277-1**	40S ribosomal protein S13	*RPS13*	-0,13129
**P26373-1; P26373-2**	60S ribosomal protein L13	*RPL13*	-0,115923
**P11940-2; P11940-1; Q9H361-1**	Polyadenylate-binding protein 1	*PABPC1; PABPC1L; PABPC3; PABPC5*	-0,104322
**P23246-1; P23246-2**	Splicing factor, proline- and glutamine-rich	*SFPQ*	-0,0925276
**P05023-3; P05023-4; P05023-1; P05023-2**	Potassium-transporting ATPase alpha chain 1	*ATP12A; ATP1A1; ATP1A2; ATP1A3; ATP1A4; ATP4A*	-0,0509272
**P43121-1; P43121-2**	Cell surface glycoprotein MUC18	*MCAM*	-0,0497869
**P10809-1**	60 kDa heat shock protein, mitochondrial	*HSPD1*	-0,0109146
**P08865-1**	40S ribosomal protein SA	*RPSA*	-0,00227096
**P05362-1**	Intercellular adhesion molecule 1	*ICAM1*	0,0491422
**P08133-2; P08133-1**	Annexin A6	*ANXA6*	0,0784256
**P55072-1**	Transitional endoplasmic reticulum ATPase	*VCP*	0,0965568
**P06733-1; P06733-2**	Alpha-enolase; Beta-enolase	*ENO1; ENO3*	0,109603
**Q9H5V8-1; Q9H5V8-2**	CUB domain-containing protein 1	*CDCP1*	0,15317
**P13010-1**	X-ray repair cross-complementing protein 5	*XRCC5*	0,178743
**P16144-4; P16144-2; P16144-1; P16144-3; P16144-5**	Integrin beta-4	*ITGB4*	0,195634

**P60174-1; P60174-3; P60174-4**	Triosephosphate isomerase	*TPI1*	0,219759
**P11021-1**	78 kDa glucose-regulated protein	*HSPA5*	0,22906
**P62263-1**	40S ribosomal protein S14	*RPS14*	0,238854
**P35613-2; P35613-1; P35613-3; P35613-4**	Basigin	*BSG*	0,247982
**P07355-1; P07355-2; A6NMY6-1**	Annexin A2	*ANXA2; ANXA2P2*	0,267505
**O00159-3; O00159-1; O00159-2**	Unconventional myosin-Ia, -Ic	*MYO1A; MYO1C*	0,282688
**O60506-4; O60506-3; O60506-5**	Heterogeneous nuclear ribonucleoprotein Q	*SYNCRIP*	0,28509
**P05783-1**	Keratin, type I cytoskeletal 18	*KRT18; KRT35*	0,300123
**Q92841-1; Q92841-3; Q92841-2; Q92841-4**	Probable ATP-dependent RNA helicase DDX17	*DDX17*	0,305261
**P68104-1; Q5VTE0-1; P68104-2**	Elongation factor 1-alpha 1	*EEF1A1; EEF1A1P5*	0,310915
**P14625-1**	Endoplasmin	*HSP90B1; HSP90B2P*	0,311944
**Q15233-1; Q15233-2**	Non-POU domain-containing octamer-binding protein	*NONO*	0,326438
**P17844-2; P17844-1**	Probable ATP-dependent RNA helicase DDX5	*DDX5*	0,330801
**P22626-1; P22626-2**	Heterogeneous nuclear ribonucleoproteins A2/B1	*HNRNPA2B1*	0,33181
**P07195-1**	L-lactate dehydrogenase B chain	*LDHB*	0,342363
**P26641-1; P26641-2**	Elongation factor 1-gamma	*EEF1G*	0,34411
**P61978-3; P61978-1; P61978-2**	Heterogeneous nuclear ribonucleoprotein K	*HNRNPK*	0,389173
**Q08211-1**	ATP-dependent RNA helicase A	*DHX9*	0,396538
**O43707-1; O43707-2; O43707-3**	Alpha-actinin-4; Spectrin beta chain	*ACTN4; SPTBN4*	0,403416
**P11586-1**	C-1-tetrahydrofolate synthase	*MTHFD1*	0,407662
**P46087-2; P46087-1; P46087-4; P46087-3**	Probable 28S rRNA (cytosine(4447)-C(5))-methyltransferase	*NOP2*	0,448728
**Q00839-2; Q00839-1**	Heterogeneous nuclear ribonucleoprotein U	*HNRNPU*	0,500035
**P12814-2; P12814-1; P12814-3; P12814-4**	Alpha-actinin-1; Alpha-actinin-2	*ACTN1; ACTN2*	0,508819
**P21333-2; P21333-1**	Filamin-A	*FLNA*	0,539827
**P23528-1**	Cofilin-1; Cofilin-2	*CFL1; CFL2*	0,552511
**Q92598-2; Q92598-3; Q92598-1; Q92598-4**	Heat shock 70 kDa protein 4L; Heat shock protein 105 kDa	*HSPA4L; HSPH1*	0,558874
**P11142-1; P11142-2**	Heat shock cognate 71 kDa protein	*HSPA8*	0,561831
**P60709-1; P63261-1**	Actin	*ACTB; ACTG1; POTEE; POTEF; POTEI; POTEJ; POTEKP*	0,575832
**P34932-1**	Heat shock 70 kDa protein 4	*HSPA4*	0,580138
**P13639-1**	Elongation factor 2	*EEF2*	0,6048
**P50995-2; P50995-1**	Annexin A11	*ANXA11*	0,660181
**P68371-1; P04350-1**	Tubulin beta chain	*TUBB*	0,70852
**P07437-1**	Tubulin beta-4A ; beta-4B	*TUBB4A; TUBB4B*	0,70852
**P50990-2; P50990-1; P50990-3**	T-complex protein 1 subunit theta	*CCT8*	0,726359
**Q15366-7; Q15366-8; Q15366-4; Q15366-5; Q15366-6; Q15366-3; Q15366-1; Q15366-2**	Poly(rC)-binding protein 2; Poly(rC)-binding protein 3	*PCBP2; PCBP3*	0,72642
**O00571-1; O00571-2; O15523-2; O15523-1**	ATP-dependent RNA helicase DDX3X, DDX3Y, DDX4	*DDX3X; DDX3Y; DDX4*	0,73737
**P21980-1; P21980-2**	Protein-glutamine gamma-glutamyltransferase 2	*TGM2*	0,76894
**P68363-1; P68363-2**	Tubulin alpha-1B chain	*TUBA1B; TUBA4B*	0,769793
**P07900-1; P07900-2**	Heat shock protein HSP 90-alpha	*HSP90AA1; HSP90AA2P; HSP90AA5P*	0,779184
**O75369-2; O75369-9; O75369-1; O75369-8;**	Filamin-B, -C	*FLNB; FLNC*	0,8168

**O75369-6; O75369-3; O75369-7; O75369-5; O75369-4**			
**P08238-1**	Heat shock protein HSP 90-beta	*HSP90AB1; HSP90AB3P*	0,85547
**P62917-1**	60S ribosomal protein L8	*RPL8*	0,882562
**P23396-1; P23396-2**	40S ribosomal protein S3	*RPS3*	0,88701
**P62805-1**	Histone H4	*HIST1H4A*	0,939429
**P0DMV9-1; P0DMV8-1; P0DMV8-2**	Heat shock 70 kDa protein 1A, -1B	*HSPA1A; HSPA1B*	0,963939
**P49748-2; P49748-1; P49748-3**	Very long-chain specific acyl-CoA dehydrogenase, mitochondrial	*ACADVL*	1,02287
**P26038-1**	Moesin; Radixin	*MSN; RDX*	1,11444
**P35579-1; P35579-2**	Myosin	*MYH1; MYH11; MYH13; MYH2; MYH3; MYH4; MYH6; MYH7; MYH7B; MYH8; MYH9*	1,11927
**P41091-1; Q2VIR3-1; Q2VIR3-2**	Eukaryotic translation initiation factor 2 subunit 3	*EIF2S3; EIF2S3L*	1,17002
**P00558-1; P00558-2**	Phosphoglycerate kinase 1, 2	*PGK1; PGK2*	1,23143
**Q99878-1; Q96KK5-1; Q9BTM1-1; Q16777-1; Q6FI13-1; P20671-1; P0C0S8-1; Q9BTM1-2**	Histone H2A type 1, 1-D; 1-H; 1-J; 2-A; 2-C; H2A.J	*H2AFJ; HIST1H2AD ; HIST1H2AG ; HIST1H2AH ; HIST1H2AJ; HIST2H2AA 3; HIST2H2AC*	1,31656
**P14618-1; P14618-3; P14618-2**	Pyruvate kinase	*PKLR; PKM*	1,61832
**P40926-1; P40926-2**	Malate dehydrogenase, mitochondrial	*MDH2*	1,70708
**Q06830-1**	Peroxiredoxin-1	*PRDX1*	1,79851
**P62979-1; P62987-1; P0CG47-1; P0CG48-1**	40S ribosomal protein S27a; 60S ribosomal protein L40	*RPS27A; UBA52; UBB; UBC*	2,17798
**P04075-1; P04075-2**	Fructose-bisphosphate aldolase A	*ALDOA*	2,26538
**P04406-1; P04406-2**	Glyceraldehyde-3-phosphate dehydrogenase	*GAPDH; GAPDHS*	2,56741
**P32119-1**	Peroxiredoxin-2	*PRDX2*	3,82495
			**SW480 F59L/ SW480 NTC**
**P05388-1; P05388-2; Q8NHW5-1**	60S acidic ribosomal protein P0; 60S acidic ribosomal protein P0-like	*RPLP0; RPLP0P6*	-1,13022
**P46777-1**	60S ribosomal protein L5	*RPL5*	-1,01215
**Q09666-1**	AHNAK; AHNAK2	*AHNAK; AHNAK2*	-0,953121
**P27816-1; P27816-6; P27816-2; P27816-4**	Microtubule-associated protein 4	*MAP4*	-0,840618
**Q7KZF4-1**	Staphylococcal nuclease domain-containing protein 1	*SND1*	-0,617826
**P05362-1**	Intercellular adhesion molecule 1	*ICAM1*	-0,541749
**Q9UQ80-1; Q9UQ80-2**	Proliferation-associated protein 2G4	*PA2G4*	-0,4693
**P68104-1; Q5VTE0-1; P68104-2**	Elongation factor 1-alpha 1; Putative elongation factor 1-alpha-like 3	*EEF1A1; EEF1A1P5*	-0,448299
**P16401-1**	Histone H1.5	*HIST1H1B*	-0,41828
**Q14103-4; Q14103-3; Q14103-2; Q14103-1**	Heterogeneous nuclear ribonucleoprotein D0	*HNRNPD*	-0,400299
**P30050-1; P30050-2**	60S ribosomal protein L12	*RPL12*	-0,399818
**P62917-1**	60S ribosomal protein L8	*RPL8*	-0,367553
**P16403-1; P10412-1**	Histone H1.1; Histone H1.2; Histone H1.4; Histone H1t	*HIST1H1A; HIST1H1C; HIST1H1E; HIST1H1T*	-0,283334
**P10809-1**	60 kDa heat shock protein, mitochondrial	*HSPD1*	-0,280466
**P21333-2; P21333-1**	Filamin-A	*FLNA*	-0,221981
**P36578-1**	60S ribosomal protein L4	*RPL4*	-0,160969

**P51114-2; P51114-1; P51114-3**	Fragile X mental retardation protein 1; Fragile X mental retardation syndrome-related protein 1	*FMR1; FXR1*	-0,100035
**P06748-2; P06748-1; P06748-3**	Nucleophosmin	*NPM1*	-0,0969905
**P11166-1**	Solute carrier family 2, facilitated glucose transporter member 1	*SLC2A1*	-0,0643709
**Q07020-1; Q07020-2**	60S ribosomal protein L18	*RPL18*	-0,0398346
**Q92841-1; Q92841-3; Q92841-2; Q92841-4**	Probable ATP-dependent RNA helicase DDX17	*DDX17*	-0,0347906
**Q12906-5; Q12906-4; Q12906-2; Q12906-6; Q12906-3; Q12906-1; Q12906-7**	Interleukin enhancer-binding factor 3	*ILF3; STRBP*	0,0146911
**P16144-4; P16144-2; P16144-1; P16144-3; P16144-5**	Integrin beta-4	*ITGB4*	0,0659784
**P26373-1; P26373-2**	60S ribosomal protein L13	*RPL13*	0,083408
**P68363-1; P68363-2**	Tubulin alpha-1B chain	*TUBA1B; TUBA4B*	0,0897768
**P07900-1; P07900-2**	Heat shock protein HSP 90-alpha; -alpha A2	*HSP90AA1; HSP90AA2P ; HSP90AA5P*	0,106045
**P06733-1; P06733-2**	Alpha-enolase; Beta-enolase	*ENO1; ENO3*	0,113265
**P68371-1; P04350-1**	Tubulin beta-4A ; beta-4B	*TUBB4A; TUBB4B*	0,17827
**Q9UHD8-7; Q9UHD8-2; Q9UHD8-5; Q9UHD8-1; Q9UHD8-3; Q9UHD8-4; Q9UHD8-9; Q9UHD8-8**	Septin-9	*SEPT9*	0,181982
**P62263-1**	40S ribosomal protein S14	*RPS14*	0,190176
**P26641-1; P26641-2**	Elongation factor 1-gamma	*EEF1G*	0,214401
**P05023-3; P05023-4; P05023-1; P05023-2**	Potassium-transporting ATPase alpha chain 1	*ATP12A; ATP1A1; ATP1A2; ATP1A3; ATP1A4; ATP4A*	0,240457
**P84098-1**	60S ribosomal protein L19	*RPL19*	0,283742
**P46087-2; P46087-1; P46087-4; P46087-3**	Probable 28S rRNA (cytosine(4447)-C(5))-methyltransferase	*NOP2*	0,284315
**P07355-1; P07355-2; A6NMY6-1**	Annexin A2	*ANXA2; ANXA2P2*	0,28788
**P29401-1; P29401-2**	Transketolase	*TKT*	0,30568
**O43707-1; O43707-2; O43707-3**	Alpha-actinin-4; Spectrin beta chain	*ACTN4; SPTBN4*	0,312668
**P23396-1; P23396-2**	40S ribosomal protein S3	*RPS3*	0,324636
**Q00839-2; Q00839-1**	Heterogeneous nuclear ribonucleoprotein U	*HNRNPU*	0,325904
**P08133-2; P08133-1**	Annexin A6	*ANXA6*	0,347591
**P11586-1**	C-1-tetrahydrofolate synthase	*MTHFD1*	0,375882
**P43121-1; P43121-2**	Cell surface glycoprotein MUC18	*MCAM*	0,38676
**P61978-3; P61978-1; P61978-2**	Heterogeneous nuclear ribonucleoprotein K	*HNRNPK*	0,413188
**P05198-1**	Eukaryotic translation initiation factor 2 subunit 1	*EIF2S1*	0,422889
**P11142-1; P11142-2**	Heat shock cognate 71 kDa protein	*HSPA8*	0,428196
**P52272-2; P52272-1**	Heterogeneous nuclear ribonucleoprotein M	*HNRNPM*	0,442281
**P35579-1; P35579-2**	Myosin	*MYH1; MYH11; MYH13; MYH2; MYH3; MYH4; MYH6; MYH7; MYH7B; MYH8; MYH9*	0,453581
**P23246-1; P23246-2**	Splicing factor, proline- and glutamine-rich	*SFPQ*	0,48784
**P05783-1**	Keratin, type I cytoskeletal 18	*KRT18; KRT35*	0,508769
**P12814-2; P12814-1; P12814-3; P12814-4**	Alpha-actinin-1; Alpha-actinin-2	*ACTN1; ACTN2*	0,529665

**P22626-1; P22626-2**	Heterogeneous nuclear ribonucleoproteins A2/B1	*HNRNPA2B 1*	0,564048
**O00159-3; O00159-1; O00159-2**	Unconventional myosin-Ia; -Ic	*MYO1A; MYO1C*	0,590048
**P13639-1**	Elongation factor 2	*EEF2*	0,629461
**P35613-2; P35613-1; P35613-3; P35613-4**	Basigin	*BSG*	0,666584
**P21980-1; P21980-2**	Protein-glutamine gamma-glutamyltransferase 2	*TGM2*	0,693221
**O60506-4; O60506-3; O60506-5**	Heterogeneous nuclear ribonucleoprotein Q	*SYNCRIP*	0,721032
**P60709-1; P63261-1**	Actin	*ACTB; ACTG1; POTEE; POTEF; POTEI; POTEJ; POTEKP*	0,77215
**Q08211-1**	ATP-dependent RNA helicase A	*DHX9*	0,807751
**O75369-2; O75369-9; O75369-1; O75369-8; O75369-6; O75369-3; O75369-7; O75369-5; O75369-4**	Filamin-B; Filamin-C	*FLNB; FLNC*	0,842432
**P04406-1; P04406-2**	Glyceraldehyde-3-phosphate dehydrogenase	*GAPDH; GAPDHS*	0,917602
**P14618-1; P14618-3; P14618-2**	Pyruvate kinase	*PKLR; PKM*	0,979855
**P62805-1**	Histone H4	*HIST1H4A*	1,11757
**P15311-1**	Ezrin	*EZR*	1,30473
**P26038-1**	Moesin; Radixin	*MSN; RDX*	1,38244
**Q99878-1; Q96KK5-1; Q9BTM1-1; Q16777-1; Q6FI13-1; P20671-1; P0C0S8-1; Q9BTM1-2**	Histone H2A type 1, 1-D; 1-H; 1-J; 2-A; 2-C; H2A.J	*H2AFJ; HIST1H2AD ; HIST1H2AG ; HIST1H2AH ; HIST1H2AJ; HIST2H2AA 3; HIST2H2AC*	1,83753
			**SW620 F52L/ SW620 NTC**
**P23229-4; P23229-2; P23229-5; P23229-3; P23229-9; P23229-6; P23229-1; P23229-7**	Integrin alpha-6	*ITGA6*	-2,23396
**P32969-1**	60S ribosomal protein L9	*RPL9*	-2,02289
**P05388-1; P05388-2; Q8NHW5-1**	60S acidic ribosomal protein P0; 60S acidic ribosomal protein P0-like	*RPLP0; RPLP0P6*	-2,02148
**P62913-2; P62913-1**	60S ribosomal protein L11	*RPL11*	-1,81988
**P16401-1**	Histone H1.5	*HIST1H1B*	-1,7514
**P16403-1; P10412-1**	Histone H1.1; H1.2; H1.4; H1t	*HIST1H1A; HIST1H1C; HIST1H1E; HIST1H1T*	-1,69603
**P11142-1; P11142-2**	Heat shock cognate 71 kDa protein	*HSPA8*	-1,679
**P31948-1; P31948-2; P31948-3**	Stress-induced-phosphoprotein 1	*STIP1*	-1,66551
**P46777-1**	60S ribosomal protein L5	*RPL5*	-1,65502
**Q14103-4; Q14103-3; Q14103-2; Q14103-1**	Heterogeneous nuclear ribonucleoprotein D0	*HNRNPD*	-1,653
**P35613-2; P35613-1; P35613-3; P35613-4**	Basigin	*BSG*	-1,65057
**Q16555-2; Q16555-1**	Dihydropyrimidinase-related protein 1, 2, 3	*CRMP1; DPYSL2; DPYSL3*	-1,64699
**P08195-2; P08195-3; P08195-1; P08195-4**	4F2 cell-surface antigen heavy chain	*SLC3A2*	-1,63265
**P30050-1; P30050-2**	60S ribosomal protein L12	*RPL12*	-1,52446
**P62851-1**	40S ribosomal protein S25	*RPS25*	-1,49801

**P62826-1**	GTP-binding nuclear protein Ran	*RAN*	-1,49631
**P27816-1; P27816-6; P27816-2; P27816-4**	Microtubule-associated protein 4	*MAP4*	-1,47485
**P11940-2; P11940-1; Q9H361-1**	Polyadenylate-binding protein	*PABPC1; PABPC1L; PABPC3; PABPC5*	-1,45818
**Q9UQ80-1; Q9UQ80-2**	Proliferation-associated protein 2G4	*PA2G4*	-1,45227
**Q9NSD9-1; Q9NSD9-2**	Phenylalanine--tRNA ligase beta subunit	*FARSB*	-1,45018
**P68363-1; P68363-2**	Tubulin alpha-1B chain	*TUBA1B; TUBA4B*	-1,42299
**P46940-1**	Ras GTPase-activating-like protein IQGAP1	*IQGAP1*	-1,41822
**P35637-2; P35637-1; Q92804-2; Q92804-1**	RNA-binding protein FUS	*FUS; TAF15*	-1,41505
**P36578-1**	60S ribosomal protein L4	*RPL4*	-1,41464
**P35221-1; P35221-2; P35221-3**	Catenin alpha-1; alpha-3	*CTNNA1; CTNNA3*	-1,3952
**P05023-3; P05023-4; P05023-1; P05023-2**	Potassium-transporting ATPase alpha chain	*ATP12A; ATP1A1; ATP1A2; ATP1A3; ATP1A4; ATP4A*	-1,33462
**Q09666-1**	AHNAK; AHNAK2	*AHNAK; AHNAK2*	-1,30513
**P09327-1**	Advillin; Villin-1	*AVIL; VIL1*	-1,27089
**P41091-1; Q2VIR3-1; Q2VIR3-2**	Eukaryotic translation initiation factor 2 subunit 3	*EIF2S3; EIF2S3L*	-1,25852
**P61247-1**	40S ribosomal protein S3a	*RPS3A*	-1,25729
**P08865-1**	40S ribosomal protein SA	*RPSA*	-1,24844
**Q7L1Q6-2; Q7L1Q6-1; Q7L1Q6-4; Q7L1Q6-3**	Basic leucine zipper and W2 domain-containing protein 1	*BZW1*	-1,24772
**P11166-1**	Solute carrier family 2, facilitated glucose transporter member 1	*SLC2A1*	-1,24752
**P68104-1; Q5VTE0-1; P68104-2**	Elongation factor 1-alpha 1	*EEF1A1; EEF1A1P5*	-1,21703
**P49588-1; P49588-2**	Alanine--tRNA ligase	*AARS*	-1,21183
**O76021-1**	Ribosomal L1 domain-containing protein 1	*RSL1D1*	-1,20809
**P12956-1; P12956-2**	X-ray repair cross-complementing protein 6	*XRCC6*	-1,205
**Q15758-1; Q15758-3; Q15758-2**	Neutral amino acid transporter B(0)	*SLC1A5*	-1,19929
**Q7KZF4-1**	Staphylococcal nuclease domain-containing protein 1	*SND1*	-1,17894
**Q9UHD8-7; Q9UHD8-2; Q9UHD8-5; Q9UHD8-1; Q9UHD8-3; Q9UHD8-4; Q9UHD8-9; Q9UHD8-8**	Septin-9	*SEPT9*	-1,16432
**P61353-1**	60S ribosomal protein L27	*RPL27*	-1,15492
**P62249-1**	40S ribosomal protein S16	*RPS16*	-1,14186
**P13010-1**	X-ray repair cross-complementing protein 5	*XRCC5*	-1,13918
**P08238-1**	Heat shock protein HSP 90-beta	*HSP90AB1; HSP90AB3P*	-1,13479
**O43707-1; O43707-2; O43707-3**	Alpha-actinin-4; Spectrin beta chain, non-erythrocytic 4	*ACTN4; SPTBN4*	-1,12383
**P15559-3; P15559-2; P15559-1**	NAD(P)H dehydrogenase [quinone] 1	*NQO1*	-1,06322
**Q92616-1**	Translational activator GCN1	*GCN1L1*	-1,06198
**P16444-1**	Dipeptidase 1	*DPEP1*	-1,06092
**P06733-1; P06733-2**	Alpha-enolase; Beta-enolase	*ENO1; ENO3*	-1,06055
**P62888-1**	60S ribosomal protein L30	*RPL30*	-1,05258
**P25705-1; P25705-2; P25705-3**	ATP synthase subunit alpha, mitochondrial	*ATP5A1*	-1,04793
**Q08211-1**	ATP-dependent RNA helicase A	*DHX9*	-1,04449

**P07900-1; P07900-2**	Heat shock protein HSP 90-alpha; -alpha A2; -alpha A5	*HSP90AA1; HSP90AA2P ; HSP90AA5P*	-1,04098
**P00558-1; P00558-2**	Phosphoglycerate kinase 1; 2	*PGK1; PGK2*	-1,03847
**P50991-1; P50991-2**	T-complex protein 1 subunit delta	*CCT4*	-1,03699
**P27824-1; P27824-2; P27824-3**	Calnexin	*CANX*	-1,01332
**P05141-1; P12236-1**	ADP/ATP translocase	*SLC25A31; SLC25A4; SLC25A5; SLC25A6*	-1,00921
**P12814-2; P12814-1; P12814-3; P12814-4**	Alpha-actinin-1; -2	*ACTN1; ACTN2*	-1,00211
**P50990-2; P50990-1; P50990-3**	T-complex protein 1 subunit theta	*CCT8*	-0,996261
**P61221-1**	ATP-binding cassette sub-family E member 1	*ABCE1*	-0,980792
**P10809-1**	60 kDa heat shock protein, mitochondrial	*HSPD1*	-0,980427
**Q15366-7; Q15366-8; Q15366-4; Q15366-5; Q15366-6; Q15366-3; Q15366-1; Q15366-2**	Poly(rC)-binding protein 2; Poly(rC)-binding protein 3	*PCBP2; PCBP3*	-0,959253
**Q12906-5; Q12906-4; Q12906-2; Q12906-6; Q12906-3; Q12906-1; Q12906-7**	Interleukin enhancer-binding factor 3	*ILF3; STRBP*	-0,952051
**Q9Y5B9-1**	FACT complex subunit SPT16	*SUPT16H*	-0,951454
**P11021-1**	78 kDa glucose-regulated protein	*HSPA5*	-0,949227
**P26641-1; P26641-2**	Elongation factor 1-gamma	*EEF1G*	-0,94274
**Q86VP6-1; Q86VP6-2**	Cullin-associated NEDD8-dissociated protein 1; Cullin-associated NEDD8-dissociated protein 2	*CAND1; CAND2*	-0,92571
**Q02878-1**	60S ribosomal protein L6	*RPL6*	-0,923298
**P14625-1**	Endoplasmin	*HSP90B1; HSP90B2P*	-0,888394
**P26373-1; P26373-2**	60S ribosomal protein L13	*RPL13*	-0,875329
**P08559-3; P08559-1; P08559-2; P08559-4**	Pyruvate dehydrogenase E1 component subunit alpha	*PDHA1*	-0,871008
**P00367-1; P00367-3; P00367-2; P49448-1**	Glutamate dehydrogenase 1, 2	*GLUD1; GLUD2*	-0,859208
**P62277-1**	40S ribosomal protein S13	*RPS13*	-0,843895
**P62979-1; P62987-1; P0CG47-1; P0CG48-1**	40S ribosomal protein S27a; 60S ribosomal protein L40	*RPS27A; UBA52; UBB; UBC*	-0,838682
**P62753-1**	40S ribosomal protein S6	*RPS6*	-0,833891
**P46776-1**	60S ribosomal protein L27a	*RPL27A*	-0,818685
**P23526-1; P23526-2**	Adenosylhomocysteinase	*AHCY*	-0,809331
**P49368-2; P49368-1**	T-complex protein 1 subunit gamma	*CCT3*	-0,808937
**Q00839-2; Q00839-1**	Heterogeneous nuclear ribonucleoprotein U	*HNRNPU*	-0,805815
**P13639-1**	Elongation factor 2	*EEF2*	-0,789191
**P49748-2; P49748-1; P49748-3**	Very long-chain specific acyl-CoA dehydrogenase, mitochondrial	*ACADVL*	-0,784798
**P68371-1; P04350-1**	Tubulin beta-4A; beta-4B	*TUBB4A; TUBB4B*	-0,782255
**Q07020-1; Q07020-2**	60S ribosomal protein L18	*RPL18*	-0,778356
**P11586-1**	C-1-tetrahydrofolate synthase	*MTHFD1*	-0,773472
**P23396-1; P23396-2**	40S ribosomal protein S3	*RPS3*	-0,763713
**P50995-2; P50995-1**	Annexin A11	*ANXA11*	-0,756993
**P21333-2; P21333-1**	Filamin-A	*FLNA*	-0,75164
**P35579-1; P35579-2**	Myosin	*MYH1; MYH11; MYH13;*	-0,75164

		MYH2; MYH3; MYH4; MYH6; MYH7; MYH7B; MYH8; MYH9	
**Q92841-1; Q92841-3; Q92841-2; Q92841-4**	Probable ATP-dependent RNA helicase DDX17	*DDX17*	-0,733843
**P18124-1**	60S ribosomal protein L7	*RPL7*	-0,705171
**P62241-1**	40S ribosomal protein S8	*RPS8*	-0,704092
**P16144-4; P16144-2; P16144-1; P16144-3; P16144-5**	Integrin beta-4	*ITGB4*	-0,70171
**P08133-2; P08133-1**	Annexin A6	*ANXA6*	-0,692738
**P07355-1; P07355-2; A6NMY6-1**	Annexin A2	*ANXA2; ANXA2P2*	-0,692698
**P00505-1; P00505-2**	Aspartate aminotransferase, mitochondrial	*GOT2*	-0,685098
**P60709-1; P63261-1**	Actin	*ACTB; ACTG1; POTEE; POTEF; POTEI; POTEJ; POTEKP*	-0,672364
**P04406-1; P04406-2**	Glyceraldehyde-3-phosphate dehydrogenase	*GAPDH; GAPDHS*	-0,653786
**P07195-1**	L-lactate dehydrogenase B chain	*LDHB*	-0,6437
**P05783-1**	Keratin, type I cytoskeletal 18	*KRT18; KRT35*	-0,627599
**P61978-3; P61978-1; P61978-2**	Heterogeneous nuclear ribonucleoprotein K	*HNRNPK*	-0,626316
**P15311-1**	Ezrin	*EZR*	-0,624292
**P04075-1; P04075-2**	Fructose-bisphosphate aldolase A	*ALDOA*	-0,621936
**P14618-1; P14618-3; P14618-2**	Pyruvate kinase	*PKLR; PKM*	-0,616192
**O75369-2; O75369-9; O75369-1; O75369-8; O75369-6; O75369-3; O75369-7; O75369-5; O75369-4**	Filamin-B; Filamin-C	*FLNB; FLNC*	-0,600804
**P62805-1**	Histone H4	*HIST1H4A*	-0,588459
**P22234-1; P22234-2**	Multifunctional protein ADE2	*PAICS*	-0,586082
**P05198-1**	Eukaryotic translation initiation factor 2 subunit 1	*EIF2S1*	-0,580605
**P26038-1**	Moesin; Radixin	*MSN; RDX*	-0,576159
**P22626-1; P22626-2**	Heterogeneous nuclear ribonucleoproteins A2/B1	*HNRNPA2B1*	-0,533164
**P17844-2; P17844-1**	Probable ATP-dependent RNA helicase DDX5	*DDX5*	-0,527677
**Q14697-1; Q14697-2**	Neutral alpha-glucosidase AB	*GANAB*	-0,522709
**P0DMV9-1; P0DMV8-1; P0DMV8-2**	Heat shock 70 kDa protein 1A; 1B	*HSPA1A; HSPA1B*	-0,48366
**Q15149-7; Q15149-8; Q15149-9; Q15149-5; Q15149-4; Q15149-6; Q15149-3; Q15149-2; Q15149-1**	Plectin	*PLEC*	-0,472279
**Q14157-4; Q14157-1; Q14157-3; Q14157-2; Q14157-5**	Ubiquitin-associated protein 2-like	*UBAP2L*	-0,467608
**P46087-2; P46087-1; P46087-4; P46087-3**	28S rRNA (cytosine(4447)-C(5))-methyltransferase	*NOP2*	-0,429005
**P62910-1**	60S ribosomal protein L32	*RPL32*	-0,418449
**Q9NR30-2; Q9NR30-1**	ATP-dependent RNA helicase DDX50	*DDX21; DDX50*	-0,325581
			**SW620 F59L/ SW620 NTC**

**P49748-2; P49748-1; P49748-3**	Very long-chain specific acyl-CoA dehydrogenase, mitochondrial	*ACADVL*	-0,82038
**Q9NSD9-1; Q9NSD9-2**	Phenylalanine--tRNA ligase beta subunit	*FARSB*	-0,666132
**P05023-3; P05023-4; P05023-1; P05023-2**	Potassium-transporting ATPase alpha chain	*ATP12A; ATP1A1; ATP1A2; ATP1A3; ATP1A4; ATP4A*	-0,634922
**P46940-1**	Ras GTPase-activating-like protein IQGAP1	*IQGAP1*	-0,611751
**P00367-1; P00367-3; P00367-2; P49448-1**	Glutamate dehydrogenase 1, 2 (mitochondrial)	*GLUD1; GLUD2*	-0,602335
**P05388-1; P05388-2; Q8NHW5-1**	60S acidic ribosomal protein P0	*RPLP0; RPLP0P6*	-0,599081
**P08195-2; P08195-3; P08195-1; P08195-4**	4F2 cell-surface antigen heavy chain	*SLC3A2*	-0,556371
**P68104-1; Q5VTE0-1; P68104-2**	Elongation factor 1-alpha 1	*EEF1A1; EEF1A1P5*	-0,556126
**P35221-1; P35221-2; P35221-3**	Catenin alpha-1; Catenin alpha-3	*CTNNA1; CTNNA3*	-0,542681
**P16401-1**	Histone H1.5	*HIST1H1B*	-0,52276
**P05141-1; P12236-1**	ADP/ATP translocase	*SLC25A31; SLC25A4; SLC25A5; SLC25A6*	-0,50683
**P35579-1; P35579-2**	Myosin	*MYH1; MYH11; MYH13; MYH2; MYH3; MYH4; MYH6; MYH7; MYH7B; MYH8; MYH9*	-0,495537
**P16403-1; P10412-1**	Histone H1.1; H1.2; H1.4; H1t	*HIST1H1A; HIST1H1C; HIST1H1E; HIST1H1T*	-0,494741
**P49368-2; P49368-1**	T-complex protein 1 subunit gamma	*CCT3*	-0,488144
**Q9UQ80-1; Q9UQ80-2**	Proliferation-associated protein 2G4	*PA2G4*	-0,462521
**Q09666-1**	AHNAK; AHNAK2	*AHNAK; AHNAK2*	-0,459673
**P50990-2; P50990-1; P50990-3**	T-complex protein 1 subunit theta	*CCT8*	-0,459505
**P62424-1**	60S ribosomal protein L7a	*RPL7A*	-0,451835
**P07900-1; P07900-2**	Heat shock protein HSP 90-alpha; -alpha A2; -alpha A5	*HSP90AA1; HSP90AA2P ; HSP90AA5P*	-0,413678
**P27816-1; P27816-6; P27816-2; P27816-4**	Microtubule-associated protein 4	*MAP4*	-0,371018
**P14625-1**	Endoplasmin	*HSP90B1; HSP90B2P*	-0,359327
**P08865-1**	40S ribosomal protein SA	*RPSA*	-0,341568
**P46777-1**	60S ribosomal protein L5	*RPL5*	-0,341343
**P30050-1; P30050-2**	60S ribosomal protein L12	*RPL12*	-0,326529
**P11940-2; P11940-1; Q9H361-1**	Polyadenylate-binding protein	*PABPC1; PABPC1L; PABPC3; PABPC5*	-0,310057
**P36578-1**	60S ribosomal protein L4	*RPL4*	-0,302183
**P06733-1; P06733-2**	Alpha-enolase; Beta-enolase	*ENO1; ENO3*	-0,211946
**Q9UHD8-7; Q9UHD8-2; Q9UHD8-5; Q9UHD8-1; Q9UHD8-3; Q9UHD8-4; Q9UHD8-9; Q9UHD8-8**	Septin-9	*SEPT9*	-0,200563

**P00505-1; P00505-2**	Aspartate aminotransferase, mitochondrial	*GOT2*	-0,187162
**P46776-1**	60S ribosomal protein L27a	*RPL27A*	-0,186352
**P62913-2; P62913-1**	60S ribosomal protein L11	*RPL11*	-0,168496
**P10809-1**	60 kDa heat shock protein, mitochondrial	*HSPD1*	-0,144494
**P13639-1**	Elongation factor 2	*EEF2*	-0,144052
**P62241-1**	40S ribosomal protein S8	*RPS8*	-0,108487
**Q14697-1; Q14697-2**	Neutral alpha-glucosidase AB	*GANAB*	-0,0983958
**P18124-1**	60S ribosomal protein L7	*RPL7*	-0,0812957
**P60174-1; P60174-3; P60174-4**	Triosephosphate isomerase	*TPI1*	-0,0689157
**P61353-1**	60S ribosomal protein L27	*RPL27*	-0,0418087
**P41091-1; Q2VIR3-1; Q2VIR3-2**	Eukaryotic translation initiation factor 2 subunit 3	*EIF2S3; EIF2S3L*	-0,0338027
**P62851-1**	40S ribosomal protein S25	*RPS25*	-0,010197
**O60841-1**	Eukaryotic translation initiation factor 5B	*EIF5B*	0,0146107
**Q7L1Q6-2; Q7L1Q6-1; Q7L1Q6-4; Q7L1Q6-3**	Basic leucine zipper and W2 domain-containing protein 1	*BZW1*	0,0157278
**P68363-1; P68363-2**	Tubulin alpha-1B chain	*TUBA1B; TUBA4B*	0,0255227
**P26641-1; P26641-2**	Elongation factor 1-gamma	*EEF1G*	0,0272873
**P14618-1; P14618-3; P14618-2**	Pyruvate kinase	*PKLR; PKM*	0,0533015
**P50991-1; P50991-2**	T-complex protein 1 subunit delta	*CCT4*	0,11064
**P14314-2; P14314-1**	Glucosidase 2 subunit beta	*PRKCSH*	0,201343
**P07195-1**	L-lactate dehydrogenase B chain	*LDHB*	0,207014
**P51114-2; P51114-1; P51114-3**	Fragile X mental retardation protein 1; Fragile X mental retardation syndrome-related protein 1	*FMR1; FXR1*	0,237072
**P11586-1**	C-1-tetrahydrofolate synthase	*MTHFD1*	0,246751
**Q92616-1**	Translational activator GCN1	*GCN1L1*	0,247323
**P13010-1**	X-ray repair cross-complementing protein 5	*XRCC5*	0,262827
**P60709-1; P63261-1**	Actin	*ACTB; ACTG1; POTEE; POTEF; POTEI; POTEJ; POTEKP*	0,31447
**P23396-1; P23396-2**	40S ribosomal protein S3	*RPS3*	0,315759
**P12956-1; P12956-2**	X-ray repair cross-complementing protein 6	*XRCC6*	0,317803
**P07355-1; P07355-2; A6NMY6-1**	Annexin A2	*ANXA2; ANXA2P2*	0,344957
**Q00839-2; Q00839-1**	Heterogeneous nuclear ribonucleoprotein U	*HNRNPU*	0,347949
**P26373-1; P26373-2**	60S ribosomal protein L13	*RPL13*	0,368396
**P23229-4; P23229-2; P23229-5; P23229-3; P23229-9; P23229-6; P23229-1; P23229-7**	Integrin alpha-6	*ITGA6*	0,375563
**P68371-1; P04350-1**	Tubulin beta-4A ; beta-4B	*TUBB4A; TUBB4B*	0,408462
**P05198-1**	Eukaryotic translation initiation factor 2 subunit 1	*EIF2S1*	0,409051
**P25705-1; P25705-2; P25705-3**	ATP synthase subunit alpha, mitochondrial	*ATP5A1*	0,409141
**P08238-1**	Heat shock protein HSP 90-beta	*HSP90AB1; HSP90AB3P*	0,417675
**P04406-1; P04406-2**	Glyceraldehyde-3-phosphate dehydrogenase	*GAPDH; GAPDHS*	0,42506
**P11142-1; P11142-2**	Heat shock cognate 71 kDa protein	*HSPA8*	0,458804
**Q7KZF4-1**	Staphylococcal nuclease domain-containing protein 1	*SND1*	0,46609

**O43707-1; O43707-2; O43707-3**	Alpha-actinin-4; Spectrin beta chain, non-erythrocytic 4	*ACTN4; SPTBN4*	0,472988
**Q12906-5; Q12906-4; Q12906-2; Q12906-6; Q12906-3; Q12906-1; Q12906-7**	Interleukin enhancer-binding factor 3	*ILF3; STRBP*	0,501727
**P50995-2; P50995-1**	Annexin A11	*ANXA11*	0,523038
**P55072-1**	Transitional endoplasmic reticulum ATPase	*VCP*	0,548364
**Q06830-1**	Peroxiredoxin-1	*PRDX1*	0,623597
**P61978-3; P61978-1; P61978-2**	Heterogeneous nuclear ribonucleoprotein K	*HNRNPK*	0,639482
**P09327-1**	Advillin; Villin-1	*AVIL; VIL1*	0,647645
**Q07020-1; Q07020-2**	60S ribosomal protein L18	*RPL18*	0,694122
**P05783-1**	Keratin, type I cytoskeletal 18	*KRT18; KRT35*	0,76603
**P22626-1; P22626-2**	Heterogeneous nuclear ribonucleoproteins A2/B1	*HNRNPA2B 1*	0,854101
**P26038-1**	Moesin; Radixin	*MSN; RDX*	0,883051
**P17844-2; P17844-1**	Probable ATP-dependent RNA helicase DDX5	*DDX5*	0,910118
**P11021-1**	78 kDa glucose-regulated protein	*HSPA5*	1,05239
**Q15758-1; Q15758-3; Q15758-2**	Neutral amino acid transporter B(0)	*SLC1A5*	2,42251
**P62979-1; P62987-1; P0CG47-1; P0CG48-1**	40S ribosomal protein S27a; 60S ribosomal protein L40	*RPS27A; UBA52; UBB; UBC*	3,88048
